# Frontal network dynamics reflect neurocomputational mechanisms for reducing maladaptive biases in motivated action

**DOI:** 10.1371/journal.pbio.2005979

**Published:** 2018-10-18

**Authors:** Jennifer C. Swart, Michael J. Frank, Jessica I. Määttä, Ole Jensen, Roshan Cools, Hanneke E. M. den Ouden

**Affiliations:** 1 Donders Institute for Brain, Cognition and Behaviour, Radboud University, Nijmegen, The Netherlands; 2 Department of Cognitive, Linguistic and Psychological Sciences, Brown University, Providence, Rhode Island, United States of America; 3 Brown Institute for Brain Sciences, Brown University, Providence, Rhode Island, United States of America; 4 Department of Psychiatry, Radboud University Medical Centre, Nijmegen, The Netherlands; 5 School of Psychology, University of Birmingham, Birmingham, United Kingdom; University of Glasgow, United Kingdom of Great Britain and Northern Ireland

## Abstract

Motivation exerts control over behavior by eliciting Pavlovian responses, which can either match or conflict with instrumental action. We can overcome maladaptive motivational influences putatively through frontal cognitive control. However, the neurocomputational mechanisms subserving this control are unclear; does control entail up-regulating instrumental systems, down-regulating Pavlovian systems, or both? We combined electroencephalography (EEG) recordings with a motivational Go/NoGo learning task (*N* = 34), in which multiple Go options enabled us to disentangle selective action learning from nonselective Pavlovian responses. Midfrontal theta-band (4 Hz–8 Hz) activity covaried with the level of Pavlovian conflict and was associated with reduced Pavlovian biases rather than reduced instrumental learning biases. Motor and lateral prefrontal regions synchronized to the midfrontal cortex, and these network dynamics predicted the reduction of Pavlovian biases over and above local, midfrontal theta activity. This work links midfrontal processing to detecting Pavlovian conflict and highlights the importance of network processing in reducing the impact of maladaptive, Pavlovian biases.

## Author summary

The anticipation of reward and punishment are key drivers of behavior: we tend to take action for rewards while holding back in the face of punishment. This motivational bias might have an overall evolutionary advantage but can also work against us in specific situations. Here, we first asked whether this motivational bias relies on innate, automatic action tendencies or whether this bias might actually itself be learned. Secondly, we studied which brain processes reduce the impact of these motivational drives when they become dysfunctional. By comparing the actions of human participants to the predictions of several mathematical models, we showed that the motivational bias in action relies partly on automatic tendencies and partly on asymmetric learning from experience. We then observed that activity over the midfrontal cortex specifically increased as a function of how dysfunctional the automatic tendencies were. Additionally, this midfrontal cortex activity was functionally connected to the motor and lateral frontal cortices, which play a role in activating and inhibiting behavior. By incorporating this connectivity into the mathematical models, we showed that stronger midfrontal connectivity predicted reduced impact of dysfunctional automatic tendencies on behavior. We propose that the midfrontal cortex detects dysfunctional action tendencies and implements cognitive control by signaling across the network.

## Introduction

Potential rewards and losses are key drivers of our actions, with consequences ranging from highly desirable (e.g., raise or promotion) to unwanted (e.g., bankruptcy) and even inconceivable (the collapse of the worldwide financial market). The valence of these outcomes is particularly well known to bias our actions: whereas anticipated rewards promote taking action, anticipated losses promote holding back from taking action [1–3]. These motivational biases are often beneficial (e.g., work harder to gain a promotion and stop spending money to avoid bankruptcy), but they can also conflict with instrumental requirements imposed by the environment [4] (e.g., the need to stop sidetracking to obtain the promotion more effectively and work harder at job applications to avoid the bankruptcy). Fortunately, we are not enslaved to our motivational drives; we can often overcome our motivational biases and adapt to the environmental requirements putatively by recruiting the midfrontal cortex [5,6] as a hub in frontal control networks [7]. Here, we set out to uncover the neurocomputational mechanisms by which the midfrontal cortex reduces motivational control over our actions.

The well-established motivational biases in action [3,8–13] arise at least partly from Pavlovian mechanisms [10,13] such that appetitive conditioned cues globally promote behavioral activation and aversive conditioned cues globally inhibit behavioral activation, independently of instrumental requirements. In other words, cue valence elicits nonselective Pavlovian (in)action rather than enhancing selective instrumental responses. These Pavlovian response tendencies can be helpful in reducing computational load by shaping our actions in a hardwired manner [14]. Consequently, the Pavlovian response tendencies become disadvantageous when they are incongruent with instrumental requirements, requiring us to rely more on the relatively flexible yet slower instrumental system. We have recently demonstrated, however, that the instrumental system is susceptible to motivational valence biases as well, which are manifested as a result of biases in learning rather than directly on choice [11]. More specifically, reward outcomes are more effective in reinforcing specific Go actions and less effective in reinforcing NoGo responses. Conversely, punishment outcomes are more effective in inducing avoidance of specific Go responses that preceded them and less effective following NoGo responses. These biases are consistent with an emerging understanding of the dopaminergic mechanisms of corticostriatal plasticity [15]. Moreover, this instrumental learning bias explains a substantial portion of the behavior otherwise attributed to a Pavlovian system. Taken together, Pavlovian and instrumental learning biases complementarily contribute to the well-known motivational bias in action.

The motivational biasing of action is often adaptive but becomes maladaptive when these biases are incongruent with the environmental requirements. The midfrontal cortex is the key region that has been linked to reducing motivational biases when these biases become maladaptive [5,6], which has been assumed so far to reflect a modulation of the Pavlovian response biases. However, previous studies did not disentangle Pavlovian and instrumental learning biases, and thus the reduced motivational biases might just as well reflect a modulation of the instrumental learning biases. Moreover, the midfrontal cortex has extensively been linked to reinforcement learning signals both at time of response and feedback [16–20] and has been proposed to modulate learning in downstream target areas [21]. Therefore, it is pertinent to assess whether the midfrontal signals are related to modulations of the Pavlovian or instrumental system.

Here, we propose that the midfrontal cortex is specifically implicated in reducing motivational biases by detecting and signaling conflict between the Pavlovian and instrumental systems, relying on similar neural mechanisms as evident in classic response conflict. Classically, the midfrontal cortex has been linked to detection of response conflict [22–26], signaling the need for cognitive control to elevate the decision threshold and prevent impulsive responses [5,27–32]. In classic response conflict tasks [33], task-irrelevant features trigger prepotent responses that can conflict with the required response as signaled by the task-relevant features. During these conflict trials, oscillatory activity in the theta frequency range (4 Hz–8 Hz) increases over the midfrontal cortex [24,25], putatively reflecting the detection of conflict, and this activity is in turn predictive of behavioral performance [23]. Moreover, successful resolution of response conflict is accompanied by increased functional connectivity between the midfrontal cortex and task-related regions (most notably, the dorsolateral prefrontal cortex (PFC) and motor cortex) [23,25] thought to reflect signaling of the increased need for control in order to elevate the decision threshold accordingly [27,29,32]. We hypothesized that the midfrontal cortex similarly i) detects conflict between the Pavlovian and instrumental systems rather than modulating Pavlovian and instrumental learning biases in general and ii) signals the Pavlovian conflict to the dorsolateral PFC and motor cortex in order to facilitate instrumental behavior by preventing impulsive, Pavlovian responses yet would not be predictive of the specific required response per se.

In the current study, we employ a motivational Go/NoGo learning task with multiple Go response options [11] and concurrent electroencephalography (EEG) surface recordings to firstly disentangle nonselective Pavlovian activation from selective instrumental responses and test whether midfrontal theta activity covaries with the level of Pavlovian conflict, in line with detection of the Pavlovian conflict. Second, we assess whether the midfrontal theta responses are associated with reduced Pavlovian response biases, reduced instrumental learning biases, or both. Finally, we test whether synchronization of the dorsolateral PFC and motor cortex predicts the reduction of the motivational biases over and above the local, midfrontal theta activity, in line with conflict resolution being instantiated by signaling to downstream targets [23–25,34,35].

## Results

### Task performance: Cue and outcome valence complementarily bias motivated action

**Fig 1:**
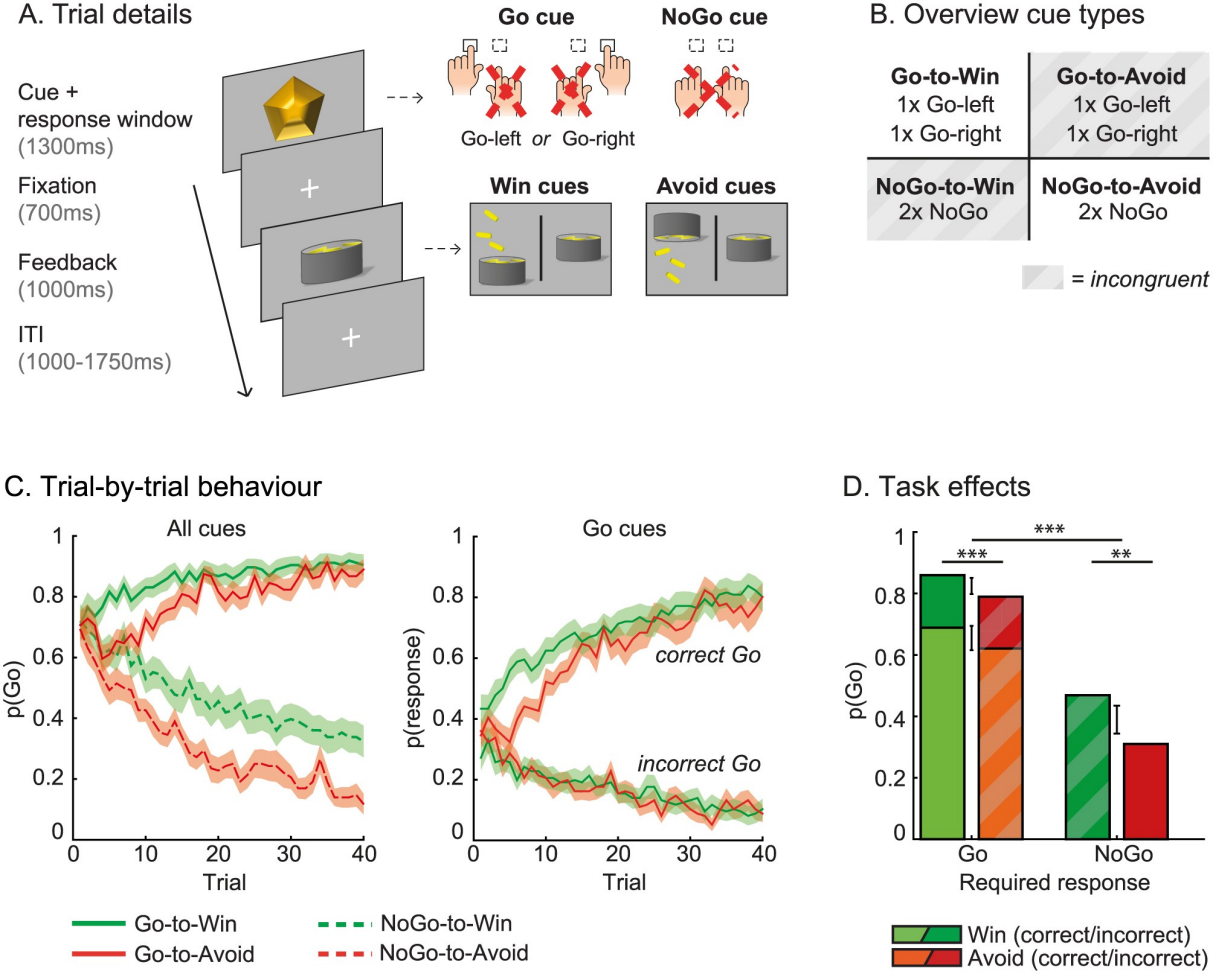
Motivational Go/NoGo learning task and performance. (a) In each trial, a cue appears on screen and response-dependent feedback follows. By trial and error, subjects should learn to press left or right (Go cues) and withhold responding (NoGo cues) during cue presentation. Feedback is probabilistic; correct responses are followed by rewards for “win” cues and neutral outcomes for “avoid” cues 80% of the time and by neutral outcomes for “win” cues and punishments for “avoid” cues otherwise. For incorrect responses, these probabilities are reversed. Rewards and punishments are visualized by money falling in and out of a basket, respectively. Image adapted from [11]. (b) Each cue has only one correct response: Go left, Go right, or NoGo. In total, there are eight different cues over which cue valence (“win” versus “avoid”) is orthogonal to the required action (Go versus NoGo). The motivationally incongruent cues, for which the Pavlovian response tendencies are opposite to the instrumental requirements, are marked in gray. (c) Average trial-by-trial behavior (shaded areas indicate the standard error of the mean) collapsed over cues of the same category. Subjects (*N* = 34) decrease incorrect Go responses (left: NoGo cues; right: Go cues) and increase correct Go responses over trials (right), illustrative of task learning. Note that only one of the Go responses (Go left/Go right) was considered correct for the Go cues, whereas all Go responses were incorrect for the NoGo cues. Notably, the initial dip in Go responses for the Go-to-Avoid cues suggests that cue valence affects Go responding once the cue valence is known, i.e., once punishment has been experienced. (d) Overall, subjects make significantly more Go responses for Go than NoGo cues. Orthogonal to the action requirements, subjects make more correct and incorrect Go responses for the “win” than “avoid” cues, which we refer to as the motivational bias. Error bars represent the standard error of the difference. ***p < 0.001. Underlying data can be found at http://hdl.handle.net/11633/di.dccn.DSC_3017033.03_624.

In the motivational Go/NoGo learning task, 34 healthy subjects needed to learn the correct responses (Go left/Go right/NoGo) by trial and error in order to gain rewards (“win” cues) and avoid losses (“avoid” cues); see Fig 1. The inclusion of multiple Go response options enables disentangling the impact of reward/punishment value on global, Pavlovian behavioral activation from that on selective, instrumental action selection and learning (see computational modeling below).

Over trials, subjects increased Go responding to the cues requiring Go versus NoGo responses (*χ*^2^(1) = 135.7, *p* < 0.001; Fig 1), indicative of task learning. Crucially, motivational valence strongly biased Go responding; subjects made more Go responses to “win” than “avoid” cues (*χ*^2^(1]) = 16.3, *p* < 0.001), independent of the Go/NoGo requirements (*χ*^2^(1) = 0.6, *p* = 0.427). This increase in Go responses was, by definition, incorrect for the NoGo cues and driven by both correct and incorrect Go responses for the Go cues (correct Go: *χ*^2^(1) = 10.8, *p* = 0.001; incorrect Go: *χ*^2^1) = 5.1, *p* = 0.024). Moreover, the accuracy of Go responses did not significantly differ for Go-to-Win and Go-to-Avoid cues (*χ*^2^(1) = 1.6, *p* = 0.200). Thus, we observed a clear effect of motivational valence on Go responding, which was driven by both correct and incorrect Go responses.

Previously, we showed that this motivational biasing of Go/NoGo responding could partly be attributed to generalized, Pavlovian response biasing and partly to biased instrumental learning of selective actions [11]. I.e., reward cues directly promoted nonselective behavioral activation, whereas reward outcomes preferentially facilitated instrumental learning for selective Go responses compared to NoGo responses. Similar Pavlovian and instrumental learning biases were observed for punishment and inaction. In the following, we disentangle the contribution of these cue-based Pavlovian response biases and outcome-based instrumental learning biases to the observed motivational bias in Go responding before turning to how they are impacted by frontal EEG signals.

**Fig 2:**
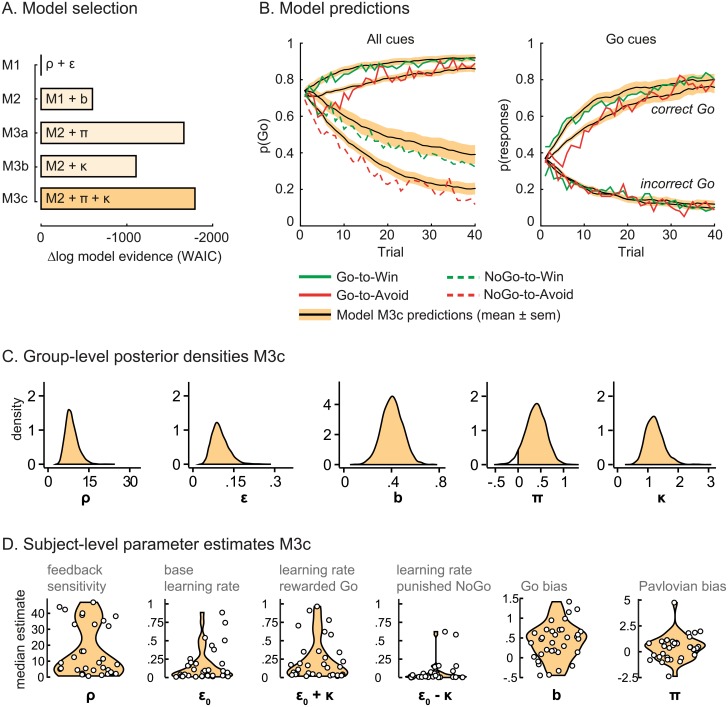
Computational modeling of Pavlovian response bias and instrumental learning bias. (a) Model evidence, relative to the simplest model, M1, favors M3c (marked by darkest color). The simplest model, M1, contains a feedback sensitivity (ρ) and learning rate (ε) parameter. Stepwise addition of the Go bias (*b*), Pavlovian bias (π), and instrumental learning bias (κ) parameter improves model fit. (b) Absolute post hoc model fit. The model predictions of winning model M3c (black lines; shaded areas indicate standard error of the mean) capture the key features of the data (colored lines); responses are learned (more Go responding for Go cues versus NoGo cues) and a motivational bias is present (more Go responding for “win” versus “avoid” cues). (c) Posterior densities of the winning model M3c. The group-level estimates are positive for the Go bias (100% samples > 0), the Pavlovian bias (95.5% samples > 0), and the instrumental learning bias (100% samples > 0). (d) Subject-level parameter estimates of the winning model M3c. The feedback sensitivity and learning rate were strongly anticorrelated over subjects (R = −0.77, *p* < 0.001) such that the impact of high feedback sensitivity was limited by a low learning rate. Subjects have increased learning rates for rewarded Go responses (ε_0_ + κ) and decreased learning rates for punished NoGo responses (ε_0_ − κ), which we refer to as the instrumental learning bias. Note that κ is added to ε_0_ prior to {0 1}-constraining; see S2 Text. Underlying data can be found at http://hdl.handle.net/11633/di.dccn.DSC_3017033.03_624. WAIC, Watanabe–Akaike Information Criterion.

We set out to disentangle the influence of Pavlovian response biases and biased instrumental learning in a computational modeling framework [11]. First, we fitted a simple reinforcement-learning model (M1) to the subjects’ choices and estimated model evidence using the Watanabe–Akaike Information Criterion (WAIC), which provides a metric of model goodness by assessing fit to the data while penalizing for model complexity. This simple model M1 included a learning rate (ε_0_) and feedback sensitivity parameter (ρ; WAIC_M1_ = 35,368; R^2^ = 33.2%). Stepwise addition of the Go bias parameter (*b;* M2), modeling a nonselective tendency towards Go responses, improved model fit (WAIC_M2_ = 34,769; R^2^ = 34.7%). Addition of the Pavlovian response bias parameter (π; M3a; WAIC_M3a_ = 33,703; R^2^ = 37.3%) and instrumental learning bias parameter (κ; M3b; WAIC_M3b_ = 34,261; R^2^ = 36.0%) further improved WAIC, indicating that Go versus NoGo responding was differentially influenced by cue and outcome valences beyond that explained by simple motor biases or instrumental learning alone. The model that best explained the task performance included both the Pavlovian response bias and instrumental learning bias parameter (M3c; WAIC_M3c_ = 33,574; R^2^ = 37.6%; Fig 2). In other words, cue and outcome valence biased behavioral activation in a complementary fashion through instantaneous, global activation and experience-dependent selective learning, respectively. See Box 1 for an overview of the model equations.

**Box 1. Overview of the behavioral computational models**

In all models, the probability of each response (*a*) is estimated based on computed action weights (*w*) using a softmax function (Eq 1, where *t* indicates the trial and *s* the state, i.e., the cue). The action weights are determined by the learned instrumental Q values (Eq 2, where ε is the learning rate and ρ the feedback sensitivity) and by a nonselective Go bias (*b*) and Pavlovian response bias (π) from, respectively, M2 and M3a onwards. Static Pavlovian values (V) were modeled once the first reward/punishment outcome had been experienced (also see S2 Text). In M3b, the instrumental learning bias (κ) is introduced (Eq 4). M3c contained both the π and κ parameters. For an elaborate description of the computational models and parameter constraints, see S2 Text.

$$p(a_{t}|s_{t})\;=\;\left[\frac{exp\;(w(a_{t},s_{t}))}{\sum_{a\prime }exp\;(w(a\prime ,s_{t}))}\right]$$ (1)

$$Q_{t}(a_{t},s_{t})\;=\;Q_{t-1}(a_{t},s_{t})+\;\varepsilon ({\rho r}_{t}-\;Q_{t-1}(a_{t},s_{t}))$$ (2)

$$\begin{array}{cc} w\left(a_{t},s_{t}\right)= & \{\begin{array}{ll} q\left(a_{t},s_{t}\right)+\;\pi V\left(s\right)+\;b & if\;a=Go\\ q\left(a_{t},s_{t}\right) & else \end{array} \end{array}$$ (3)

$$\begin{array}{cc} \varepsilon = & \{\begin{array}{ll} \varepsilon_{0}+\kappa & if\;r_{t}=1\;\&\;a=go\\ \varepsilon_{0}-\kappa & if\;r_{t}=-1\;\&\;a=nogo\\ \varepsilon_{0} & else \end{array} \end{array}$$ (4)

In the winning model, M3c, the Pavlovian bias estimates were positive at the group level (95.5% group-level samples > 0), indicating that “win” cues globally promoted Go responding while “avoid” cues globally suppressed Go responding. The instrumental learning bias estimates were positive as well (100% group-level samples > 0), indicating that reward enhanced the selective learning of Go responses (evidenced by higher learning rates for rewarded Go responses; ε_0_ + κ). Thus, subjects were more likely to repeat rewarded Go responses relative to rewarded NoGo responses. Conversely, punishment hampered the unlearning of NoGo responses (evidenced by lower learning rates for punished NoGo responses; ε_0_ **−** κ). In other words, subjects were more likely to repeat punished NoGo responses relative to repeating punished Go responses. As reported previously, the Pavlovian bias parameter decreased when including the instrumental learning bias parameter (median [25th–75th percentile] π for M3a: 0.6 {**−**0.2 1.2}; for M3c: 0.4 {**−**0.5 0.9}), suggesting that variance corresponding to the instrumental learning bias would otherwise be attributed to the Pavlovian bias parameter. The impact of the Pavlovian response bias and instrumental learning bias on behavior has distinct dynamics, however [11]; whereas the impact of the instrumental learning bias is experience dependent and develops over time, the impact of the Pavlovian bias reduces as instrumental action values are learned. Altogether, we observed a clear influence of both cue and outcome valence in biasing instrumental responding, consistent with our prior report [11].

### Midfrontal oscillatory theta power reflects Pavlovian conflict

After establishing the impact of both cue-driven (Pavlovian) and outcome-driven (instrumental learning) motivational biases, we set out to replicate the finding that midfrontal oscillatory activity in the theta frequency range (4 Hz–8 Hz) relates to reducing the motivational biasing of action [6]. Moreover, the current paradigm allows us to address specifically whether the midfrontal cortex does so by modulating the Pavlovian response bias, the instrumental learning bias, or both.

**Fig 3:**
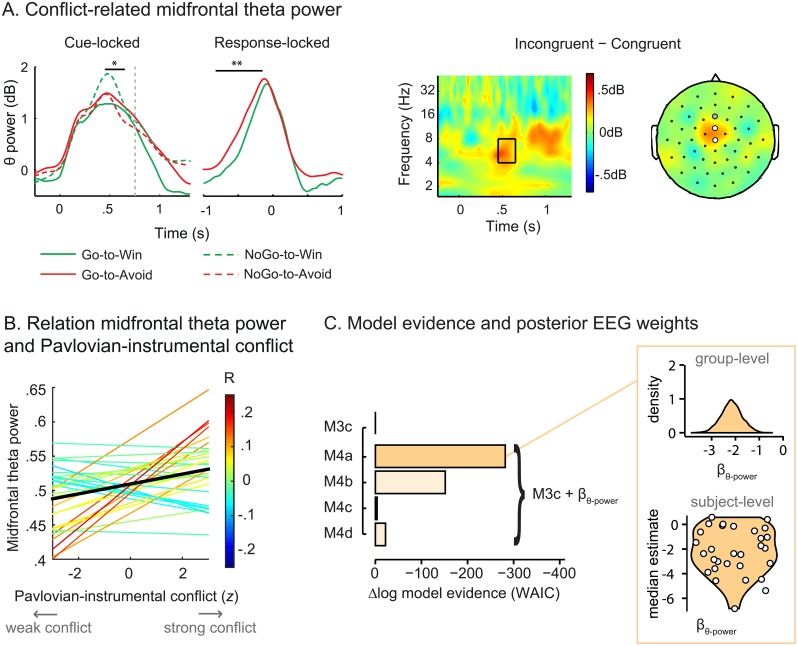
Cue-related midfrontal theta power. (a) Left: Time-wise permutation testing reveals one cue-locked (450 ms–650 ms) and response-locked (–826 ms to –150 ms) time window during which midfrontal theta power (4 Hz–8 Hz) increased for motivationally incongruent trials (NoGo-to-Win, Go-to-Avoid) relative to congruent trials (Go-to-Win, NoGo-to-Avoid). *p < 0.05; **p < 0.01. Right: Cue-locked contrast of motivational conflict. Time-frequency plot for the midfrontal channels, with the resulting time window indicated by the box. Topoplot for theta power in the resulting time window with the significant (nonsignificant) a priori midfrontal channels indicated by white (gray) discs (post hoc Bonferroni corrected alpha = 0.017). The a priori channels showing a significant cue-locked congruency effect were used in the computational models and served as seeds for the connectivity analyses. (b) Within-subject regression lines (colored) of trial-by-trial midfrontal theta power and Pavlovian–instrumental conflict according to model M3c. Across subjects (black line), there is a positive association between the level of conflict and midfrontal theta power (*p* = 0.007), putatively reflecting the detection of the conflict. Midfrontal theta power did not significantly covary with trial number (*p* = 0.94), rendering a confound of trials on task unlikely. (c) Model evidence relative to winning behavioral model M3c. Addition of the β_θ-power_ parameter improves model fit most when scaling the impact of theta power on the Pavlovian bias (M4a) rather than scaling the impact on the instrumental contribution (M4b), the balance between the Pavlovian and instrumental contribution (M4c), or the instrumental learning bias (M4d). The negative β_θ-power_ parameter estimates indicate that the conflict-related increases in midfrontal theta power were associated with reduction of the Pavlovian response bias. Underlying data can be found at http://hdl.handle.net/11633/di.dccn.DSC_3017033.03_624. WAIC, Watanabe–Akaike Information Criterion.

We reasoned that if midfrontal oscillatory theta activity relates to modulating the Pavlovian response bias [6], midfrontal theta power would increase for motivationally incongruent cues (Go-to-Avoid, NoGo-to-Win) compared to congruent cues (Go-to-Win, NoGo-to-Avoid). Because midfrontal theta has been linked to a conflict-induced adjustment of the decision threshold, preventing impulsive responses [5,27–30], we further hypothesized that increases in midfrontal theta would be particularly evident when motivational conflict is correctly resolved. Put simply, we reasoned that a control-related signal should prevail when control is successfully implemented. To this end, we specifically assessed correct trials in line with classic cognitive control studies [22–26] and report the analysis of incorrect trials in S7 Text. Time-wise permutation testing over our a priori midfrontal channels (see Methods) indeed revealed a cue-locked time window during which midfrontal theta power was significantly higher for motivationally incongruent relative to congruent cues (450 ms–650 ms; *p* = 0.024; Fig 3). Importantly, the significant time window was prior to the average response time (mean = 753 ms; range = 594 ms–1,077 ms), as might be expected for a control-related signal. Corroborating these findings, response-locked permutation testing also indicated a preresponse time window (**−**826 ms to **−**150ms; *p* = 0.002; Fig 3) during which midfrontal theta power increased for Go-to-Avoid (incongruent) trials relative to Go-to-Win (congruent) trials. Moreover, these temporally specific cue-locked and response-locked enhancements of midfrontal theta for incongruent trials were not accompanied by any converse time points in which theta power showed the reverse effect (i.e., increases for congruent relative to incongruent). Altogether, we observed a clear midfrontal theta response to motivational conflict in which theta power increased prior to correctly responding when cue valence was incongruent with the instrumental Go/NoGo requirements.

Recent theories suggest that the midfrontal cortex might be responsible for detection of conflict [22–26] and signaling the need for control to downstream targets (other cortical and subcortical sites) [23–25,34,35]. Accordingly, we reasoned that if midfrontal theta power relates to the detection of Pavlovian–instrumental conflict, the midfrontal theta signal would covary with the level of conflict. To this end, we assessed whether there was evidence for a trial-by-trial relation between the cue-locked midfrontal theta power and the level of Pavlovian–instrumental conflict. Here, we quantified Pavlovian–instrumental conflict by the extent to which instrumental values for NoGo responses were higher than those for Go responses on “win” trials and the opposite for “avoid” trials. Across subjects, this correlation was significantly positive (M3c: *t*_29_ = 2.9, *p* = 0.007; see Fig 3), supporting the trial-by-trial relation of midfrontal theta power and the level of motivational conflict. In sum, midfrontal theta power covaried with the level of motivational conflict, putatively reflecting the detection of conflict.

To assess whether the conflict-related midfrontal theta signal was indeed associated with reducing the motivational biasing of action, we extended our computational modeling approach. We employed the models developed by Cavanagh and colleagues [6] to test the following differential mechanisms by which midfrontal theta might modulate the motivational biasing of action. We tested whether midfrontal theta power altered behavior by modulating the Pavlovian response tendencies (M4a), the instrumental contribution (M4b), and/or the balance between the Pavlovian and instrumental contribution (M4c). Note that the current design is particularly well suited to differentiate between these alternative mechanisms (M4a–c), as the multiple Go options enable us to disentangle whether frontal theta facilitates selection of particular instrumental actions or rather reduces global Pavlovian (in)activation. Finally, we tested the alternative possibility that cue-related midfrontal theta power instead modulates the instrumental learning bias. I.e., midfrontal theta power at the time of the decision might affect instrumental learning that takes place thereafter. We assessed this novel hypothesis in model M4d. See Box 2 for an overview of the equations for the EEG model extension.

**Box 2. Behavioral computational models extended with trial-by-trial EEG data**

All EEG models were an extension of the behavioral model M3c; see Box 1. The β parameter scaled the impact of trial-by-trial theta power (θ_t_) on the Pavlovian bias (Eq 5; M4a), the instrumental contribution (Eq 6; M4b), the balance between the Pavlovian and instrumental contribution (Eq 7; M4c), or the instrumental learning bias (Eq 8; M4d). In model M4c, the Pavlovian bias parameter was replaced by τ, scaling the relative contribution of the Pavlovian and instrumental values. For models M5a–b, the trial-by-trial theta power estimates in Eq 5 were replaced by trial-by-trial estimates of midfrontal–lateral prefrontal phase synchrony (M5a) and midfrontal–contralateral motor phase synchrony (M5b). For an elaborate description of the computational models and parameter constraints, see S2 Text.

$$w\left(Go\prime_{t},s_{t}\right)=\{\begin{array}{ll} Q\left(Go\prime_{t},s_{t}\right)+\left(\pi +\;\beta *\theta_{t}\right)*V\left(s\right)+\;b & if\;conflict\\ Q\left(Go\prime_{t},s_{t}\right)+\;\pi V\left(s\right)+\;b & else \end{array}$$ (5)

$$\begin{array}{ll} \left(G{o^{\prime }}_{t},s_{t}\right)= & \{\begin{array}{ll} \left(1-\;\beta *\theta_{t}\right)*Q\left(G{o^{\prime }}_{t},s_{t}\right)+\pi V\left(s\right)+\;b & if\;conflict\\ Q\left(G{o^{\prime }}_{t},s_{t}\right)+\;\pi V\left(s\right)+\;b & else \end{array}\\ w\left(NoGo_{t},s_{t}\right)= & \{\begin{array}{ll} \left(1-\;\beta *\theta_{t}\right)*Q\left(NoGo_{t},s_{t}\right) & if\;conflict\\ Q\left(NoGo_{t},s_{t}\right) & else \end{array} \end{array}$$ (6)

$$\begin{array}{ll} w\left(Go\prime_{t},s_{t}\right)= & \{\begin{array}{ll} \left(1-(\tau +\;\beta *\theta_{t}\right))*Q\left(Go\prime_{t},s_{t}\right)+\left(\tau +\;\beta *\theta_{t}\right)*V\left(s\right)+\;b & if\;conflict\\ \left(1-\tau \right)*Q\left(G{o^{\prime }}_{t},s_{t}\right)+\;\tau *V\left(s\right)+\;b & else \end{array}\\ \left(NoGo_{t},s_{t}\right)= & \{\begin{array}{ll} \left(1-(\tau +\;\beta *\theta_{t}\right))*Q\left(NoGo_{t},s_{t}\right) & if\;conflict\\ \left(1-\tau \right)*Q\left(NoGo_{t},s_{t}\right) & else \end{array} \end{array}$$ (7)

$$\begin{array}{ll} \varepsilon_{rewarded\;Go}= & \{\begin{array}{ll} \left(1-\beta *\theta_{t}\right)*(\varepsilon_{0}+\kappa )+\left(\beta *\theta_{t}\right){*\varepsilon }_{0} & if\;conflict\\ \varepsilon_{0}+\kappa & else \end{array}\\ \varepsilon_{punished\;NoGo}= & \{\begin{array}{ll} \left(1-\beta *\theta_{t}\right)*(\varepsilon_{0}-\kappa )+\left(\beta *\theta_{t}\right){*\varepsilon }_{0} & if\;conflict\\ \varepsilon_{0}-\kappa & else \end{array} \end{array}$$ (8)

For model comparison, we refitted the winning base model M3c over the behavioral data of the subjects included in the EEG analyses (*N* = 30; EEG data of four subjects contained excessive noise), as model evidence can only be directly compared when estimated over identical data. When allowing midfrontal theta power to scale the impact of the Pavlovian bias, model evidence greatly increased (M4a; WAIC_M4a_ = 28,503; R^2^ = 40.5%), relative to the refitted base model M3c (WAIC_M3c, *N* = 30_ = 28,785; R^2^ = 39.8%; Fig 3), indicating that, trial-wise, midfrontal theta values are informative about the modulation of Pavlovian biases over and above what could be inferred on average across trials. There was less evidence for midfrontal theta power scaling the impact of the instrumental action values (M4b; WAIC_M4b_ = 28,634; R^2^ = 40.1%) or the balance between the Pavlovian and instrumental contribution (M4c; WAIC_M4c_ = 28,781; R^2^ = 39.5%), although both models did better than base model M3c. Although the improvements in the R^2^ values might appear small, these improvements reflect the improvement for every subject on every trial. Additionally, the impact of the additional parameters in the EEG models is restricted because i) EEG data was rejected for trials with EEG artifacts and noise (approximately 10% of trials) and ii) the EEG parameters are only modeled in half the trials (i.e., incongruent cues). Model evidence hardly increased relative to M3c when midfrontal theta power was allowed to modulate the instrumental learning bias (M4d; WAIC_M4d_ = 28,763; R^2^ = 39.8%). Thus, computational modeling indeed implicated the conflict-related midfrontal theta signals in modulation of the motivational biasing of action, for which the theta signals particularly scaled the Pavlovian response bias (M4a) rather than the instrumental learning bias (M4d).

In the winning model, M4a, the impact of midfrontal theta power on the Pavlovian bias, as assessed by β_θ-power_, has negative group-level estimates (100% samples < 0). These negative estimates indicate that midfrontal theta power relates to weaker impact of the Pavlovian response tendencies such that higher levels of theta power are associated with better ability to resolve the Pavlovian conflict. Crucially, in model M4a, midfrontal theta power reduces the Pavlovian response tendencies only under conflict between the Pavlovian response tendencies and the instrumental action values. Modeling an effect of midfrontal theta power on all trials (i.e., including motivationally congruent trials) greatly reduces model fit relative to M4a (ΔWAIC = +266), consistent with previous reports showing that midfrontal theta power relates to the decision threshold specifically on conflict trials [27–31]. Altogether, these results suggest that cue-related midfrontal theta power is associated with resolving Pavlovian–instrumental conflict rather than promoting unbiased behavior in general.

**Fig 4:**
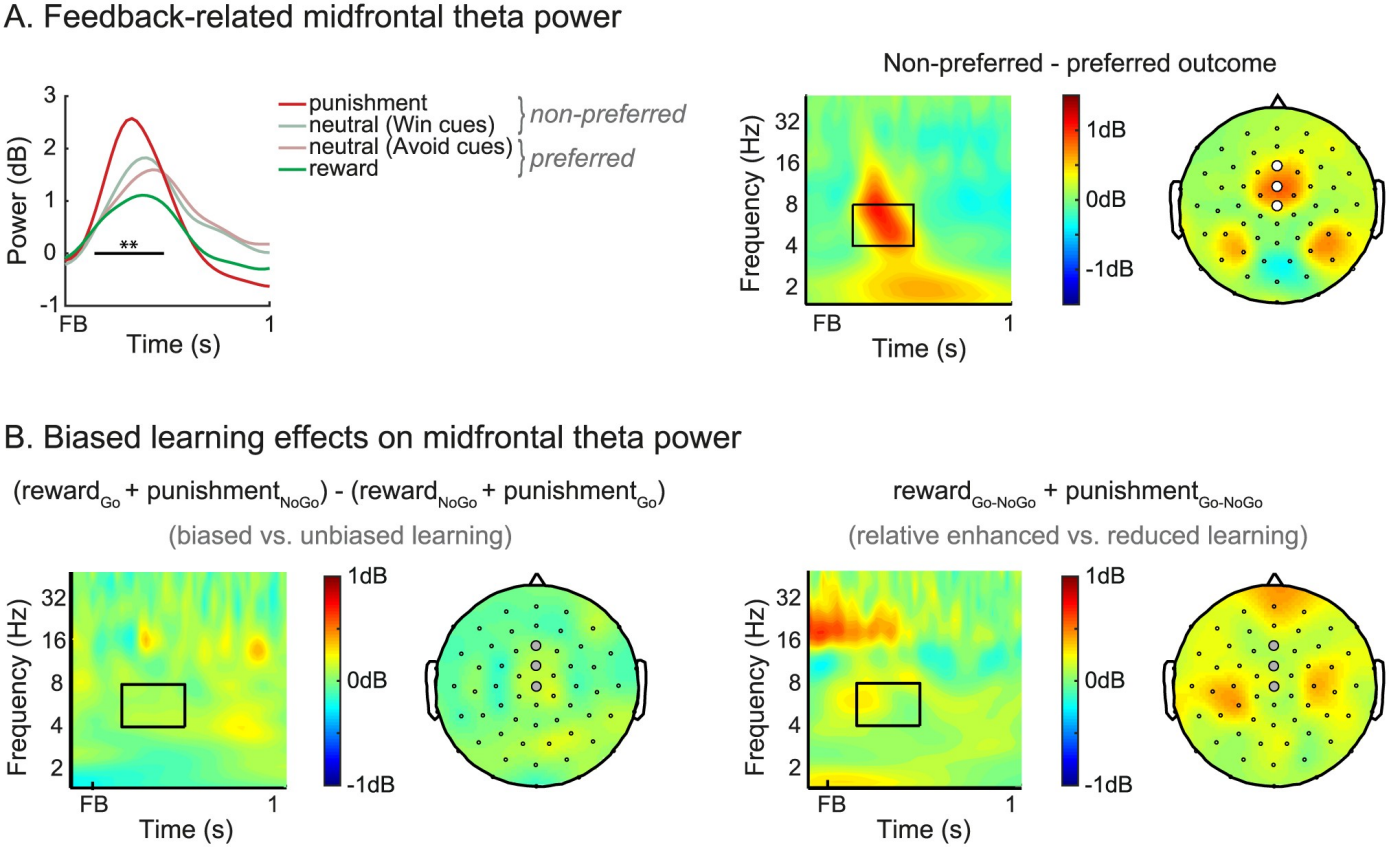
Feedback-related midfrontal theta power. (a) Left: Time-wise permutation testing reveals one feedback-locked (150 ms–467 ms) time window during which midfrontal theta power increased for nonpreferred relative to preferred outcomes. **p < 0.01. During this time window, feedback-related theta power additionally increased more for “avoid” than “win” cues (*p* < 0.001; see S5 Text). Right: Contrast for nonpreferred versus preferred outcomes. The significant (nonsignificant) a priori midfrontal channels are again indicated by white (gray) discs (post hoc Bonferroni corrected alpha = 0.017). (b) Feedback-related theta power did not vary as a function of biased learning. Left: Midfrontal theta power within the resulting time window did not significantly increase for the conditions in which we observed biased learning at the behavioral level (rewarded Go: ε_0_ + κ and punished NoGo: ε_0_ − κ) relative to their unbiased counterparts (rewarded NoGo and punished Go: ε_0_; BF_01_ = 4.9). Right: Midfrontal theta power also did not significantly vary as a function of relative magnitude of instrumental learning. Here, we contrasted rewarded Go responses (enhanced learning: ε_0_ + κ) and punished Go with rewarded NoGo and punished NoGo (decreased learning: ε_0_ − κ; BF_01_ = 3.1). Underlying data can be found at http://hdl.handle.net/11633/di.dccn.DSC_3017033.03_624.

Although the cue-related midfrontal theta power was not linked to a modulation of the instrumental learning bias (M4d), theta power at the time of outcome, when reinforcement learning would occur, could in principle modulate the learning bias nonetheless. To this end, we next assessed whether feedback-related midfrontal theta power increased differentially following actions and outcomes that are consistent with instrumental learning biases. First, we determined the optimal time window for feedback-related theta power by contrasting nonpreferred outcomes (i.e., neutral outcomes for “win” cues and punishment for “avoid” cues) and preferred outcomes (i.e., reward for “win” cues and neutral outcomes for “avoid” cues). Time-wise permutation testing using the a priori midfrontal channels revealed one time window during which midfrontal theta power was significantly higher for nonpreferred than preferred outcomes (150 ms–476 ms; *p* = 0.002; Fig 4). We then assessed whether theta power in this time window increased for the conditions in which we observed biased learning (i.e., enhanced Go learning after reward and decreased NoGo unlearning after punishment; see the Task performance: Cue and outcome valence complementarily bias motivated action section). We did not observe a significant change in midfrontal theta power for the biased learning conditions (rewarded Go: ε_0_ + κ, punished NoGo: ε_0_ **−** κ) relative to their unbiased counterparts (rewarded NoGo, punished Go; *F*_1,28_ = 1.1, *p* = 0.312, *BF*_01_ = 4.9). Alternatively, feedback-related midfrontal theta power might reflect the amount of instrumental learning that takes place. However, midfrontal theta power did not significantly differ from the conditions for which we observed relatively stronger (rewarded Go, punished Go) versus weaker (rewarded NoGo, punished NoGo) instrumental learning as observed at the behavioral level (*F*_1,28_ = 3.0, *p* = 0.095, *BF*_01_ = 3.1). Altogether, we observed well-established feedback-related modulations of midfrontal theta power [21], yet this feedback-related theta power was not linked to biased instrumental learning of Go/NoGo responses. For full analysis of the feedback-related theta power, including the neutral outcomes, we refer to S5 Text.

Taken together, cue-related midfrontal theta power reflected the level of Pavlovian conflict and was associated with reducing the Pavlovian response tendencies, whereas outcome-related midfrontal theta simply reflected feedback-valence effects and was unrelated to the biased Go/NoGo learning. Together, these results suggest that the midfrontal cortex detects Pavlovian conflict and reduces the motivational biasing of action by subsequently modulating the Pavlovian response tendencies rather than the bias in instrumental learning.

### Midfrontal network dynamics reflect the ability to overcome Pavlovian response biases

In the previous section, we established that midfrontal theta power was enhanced when motivationally driven Pavlovian response tendencies conflicted with instrumental requirements, i.e., on incongruent trials. We also showed that stronger trial-by-trial midfrontal theta activity within these incongruent trials were associated with reduced impact of the Pavlovian bias parameter rather than reduced instrumental learning biases. We next hypothesized that the midfrontal cortex might instantiate this reduction of the Pavlovian response tendencies through functional connectivity with a network of task-relevant regions, specifically the dorsolateral prefrontal and motor sites, given that increased theta phase synchrony of these regions to the midfrontal cortex has been linked to conflict processing [23–25,34,35]. Accordingly, we reasoned that motivational congruency would differentially affect the intersite phase synchrony (ISPS) between the midfrontal channels and nodes in this network. Crucially, midfrontal ISPS has been proposed to be activity dependent [36] such that target sites become more responsive to the midfrontal signals when they are more active. Therefore, we disentangled the executing motor sites (i.e., contralateral Go responses) and the nonexecuting motor sites (i.e., ipsilateral Go responses and bilateral NoGo responses) to account for putative differences in task activation due to the instantiation of overt motor responses.

**Fig 5:**
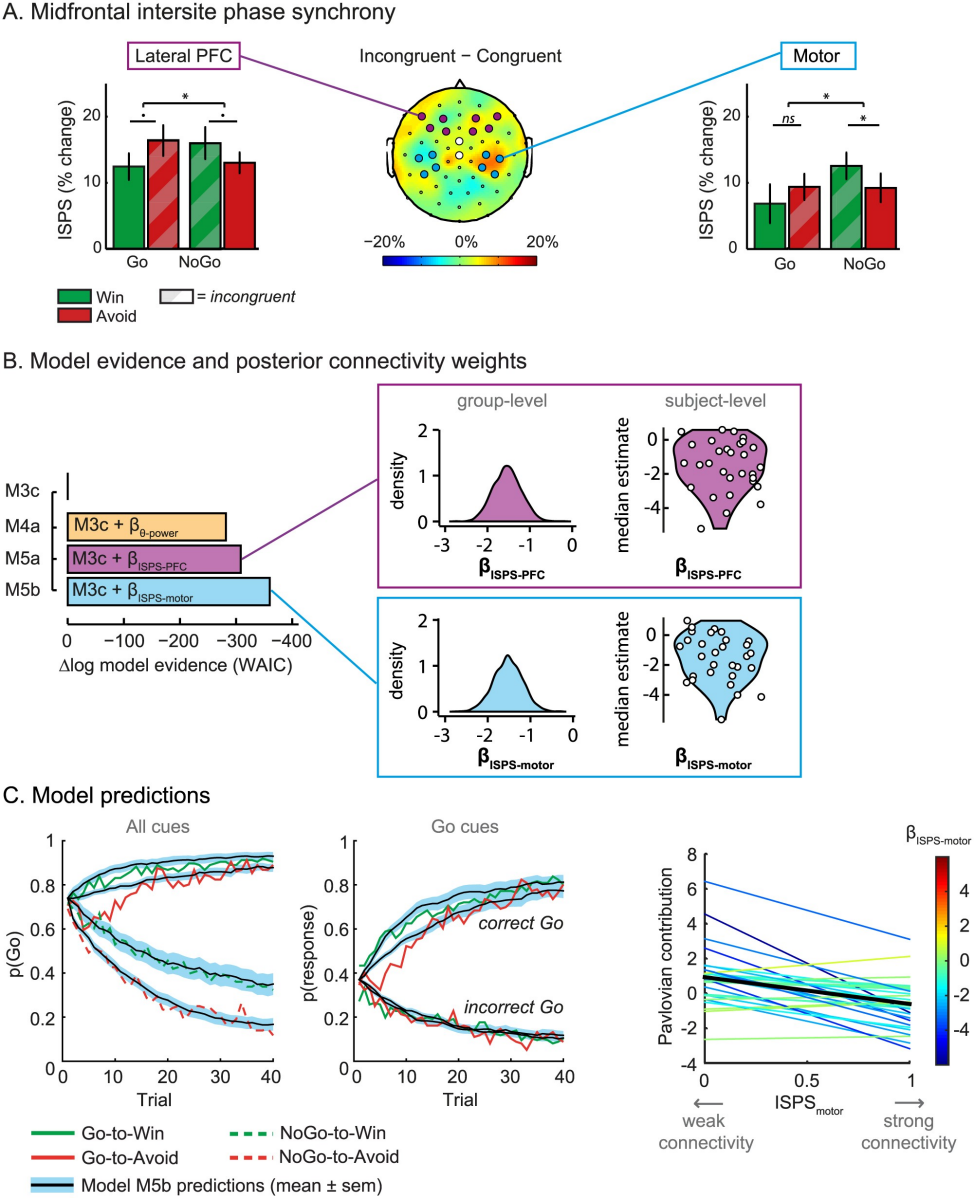
Midfrontal ISPS with lateral prefrontal and motor sites. (a) Left: Midfrontal–lateral prefrontal phase synchrony increased for motivationally incongruent cues relative to congruent cues (*p* = 0.018). *p < 0.05; •*p* < 0.1. Middle: Topographic distribution of ISPS for incongruent relative to congruent trials. The midfrontal channels showing the congruency effect of interest (see Fig 3) serve as a t-weighted seed cluster (white discs). The lateral prefrontal and motor target channels are indicated with purple and blue discs, respectively. Here, we report the nonexecuting motor channels for simplicity (see S6 Text for an in-depth discussion of the executing motor sites). Note that all subjects were right-handed, which might explain the somewhat lateralized ISPS over the motor cortex. Right: Motor–midfrontal ISPS also increased with motivational incongruency (*p* = 0.029). (b) Model evidence relative to winning behavioral model M3c. Model evidence improves even further for the models in which midfrontal–prefrontal (β_ISPS-PFC_; M5a) or midfrontal–motor (β_ISPS-motor_; M5b) phase synchrony scales the Pavlovian bias compared to local, midfrontal theta power scaling the Pavlovian bias (β_θ-power_; M4a). The novel β_ISPS-PFC_ and β_ISPS-motor_ parameter estimates are negative (group-level both: 100%), indicating the synchrony relates to reduced Pavlovian biases. (c) Left: M5b model predictions (black lines; shaded areas indicate the standard error of the mean) resemble the behavioral data (colored lines). The improvement in model predictions by the β_ISPS-motor_ parameter is particularly apparent for the NoGo cues. Right: Effect of midfrontal–motor connectivity on the Pavlovian contribution for all individuals (colored lines). Across the group (black line), stronger midfrontal–motor connectivity reflects improved ability to reduce the Pavlovian contribution. Underlying data can be found at http://hdl.handle.net/11633/di.dccn.DSC_3017033.03_624. ISPS, intersite phase synchrony; PFC, prefrontal cortex; WAIC, Watanabe–Akaike Information Criterion.

Motivational congruency indeed modulated phase synchrony between the lateral prefrontal sites and the midfrontal cluster (*F*_1,27_ = 6.3, *p* = 0.018; Fig 5), such that theta-band phase synchrony was strengthened for incongruent relative to congruent cues. This congruency effect was not significantly driven by either Go cues (Go-to-Avoid > Go-to-Win: *t*_28_ = **−**1.8, *p* = 0.089) or NoGo cues alone (NoGo-to-Win > NoGo to Avoid: *t*_28_ = 1.7, *p* = 0.093). The midfrontal–prefrontal phase synchrony did not show significant main effects of valence (*F*_1,27_ = 0.2, *p* = 0.623) or required action (*F*_1,27_ = 0.1, *p* = 0.730). Mirroring the midfrontal–prefrontal phase synchrony, phase synchrony between the midfrontal and nonexecuting motor sites also increased during incongruent cues (*F*_1,27_ = 5.3, *p* = 0.029; Fig 5) in the absence of a main effect of valence (*F*_1,27_ < 1, *p* = 0.856) and required action (*F*_1,27_ = 2.3, *p* = 0.144). The motor synchrony significantly increased with motivational conflict during NoGo trials (*t*_28_ = 2.5, *p* = 0.018, i.e., more phase synchrony on NoGo-to-Win than NoGo-to-Avoid trials) but nonsignificantly during the Go trials (*t*_28_ < 1, *p* = 0.359). Taken together, we observed clear conflict-related changes in midfrontal theta phase synchrony with both the lateral prefrontal sites and the nonexecuting motor sites.

Surprisingly, we did not observe a significant congruency effect in the executing motor sites (*t*_28_ = 1.7, *p* = 0.097), which was, if anything, in the opposite direction (congruency × motor execution: *F*_1,27_ = 10.2, *p* = 0.004; valence × required action: *F*_2,54_ = 4.0, *p* = 0.024). The absence of significant congruency effects for the Go responses seems to be in line with the cue-locked midfrontal theta power observations, in which the congruency effect also appears stronger for the NoGo than the Go cues (Fig 3A). For a more elaborate discussion of the motor ISPS results for the executing motor sites, we refer to S6 Text.

We next assessed whether the conflict-related changes in midfrontal theta phase synchrony with the lateral prefrontal (M5a) and motor (M5b) sites could also help to explain trial-by-trial choice variability related to reducing the Pavlovian response tendencies under motivational conflict. To this end, we adopted a similar modeling approach as above but using trial-by-trial synchrony estimates instead of power to scale the Pavlovian bias. Before doing so, we confirmed that the single-trial estimates replicate the trial-averaged results reported above (S7 Text). We then tested whether midfrontal–prefrontal phase synchrony could scale the impact of the Pavlovian bias for both Go responses, whereas the midfrontal–motor phase synchrony could scale the Pavlovian impact for the contralateral Go response option. Model evidence increased for both ISPS models, compared with the winning theta-power–based model (M4a; WAIC_M4a_ = 28,503), in which midfrontal–lateral prefrontal (WAIC_M5a_ = 28,477; R^2^ = 40.6%) and midfrontal–motor phase synchrony (WAIC_M5b_ = 28,425; R^2^ = 40.8%) modulated the Pavlovian bias. Although the trial-by-trial estimates of midfrontal theta power and ISPS correlated positively (correlations ranging from +0.12 to +0.62; see S7 Text), there was considerable unique variance within both measures (≥62%). Crucially, model evidence improved hardly any further when allowing the trial-by-trial estimates of midfrontal theta power to additionally modulate the Pavlovian bias in both synchrony models (both models: ΔWAIC = **−**7; WAIC_PFC_ = 28,470; WAIC_motor_ = 28,418). In other words, while theta ISPS explained behavior over and above midfrontal theta power (ΔWAIC_PFC_ = **−**33; ΔWAIC_motor_ = **−**85), midfrontal theta power barely explained any variance in addition to the phase synchrony. Thus, choices on incongruent trials were better explained when we allowed modulation of the Pavlovian response bias by intersite theta phase synchrony between the midfrontal and both task-relevant sites (lateral prefrontal and motor cluster). Alternatively, target-dependent effects of ISPS, such as lateral prefrontal synchrony modulating the goal representations and motor synchrony modulating motor excitability, reduced model evidence (S8 Text). See Fig 5 for model comparison and absolute model fit of the winning EEG model.

In this winning family of synchrony models, the novel β parameters, scaling the impact of the midfrontal–lateral prefrontal (β_PFC_; M5a) and midfrontal–motor (β_motor_; M5b) phase synchrony on the Pavlovian bias, have negative group-level estimates (both: 100% samples < 0). These negative estimates indicate that midfrontal synchrony with these task-relevant areas is related to weaker impact of the Pavlovian response tendencies, such that stronger connectivity is associated with better ability to resolve the Pavlovian conflict (Fig 5). Altogether, these results indicate that trial-by-trial theta-band synchrony within a network of task-relevant regions provides a better explanation of the ability to resolve Pavlovian conflict than theta power over the midfrontal cortex alone. This is in line with recent theories suggesting that the midfrontal cortex might be responsible for detection of the conflict and hence the need for control, whereas the implementation of control is signaled by synchrony with downstream targets (other cortical and subcortical sites) [23–25,34,35].

## Discussion

Motivation is a key driver of our actions. Here, we showed that both Pavlovian and instrumental learning mechanisms contribute to the motivational biasing of action, coupling action to reward and inaction to punishment. Theta power increased over the midfrontal cortex particularly when the prepotent, Pavlovian response tendencies conflicted with the instrumental task requirements. This conflict-related theta signal was associated with reduced Pavlovian response biases, rather than with reduced instrumental learning biases or enhanced specific instrumental responses. This conflict-related theta signal was accompanied by phase synchronization of the lateral prefrontal and motor sites to the midfrontal site, and these network dynamics predicted resolution of the Pavlovian conflict over and above local, midfrontal power.

In the motivational Go/NoGo learning tasks, subjects needed to learn to make one of two active Go responses or withhold responding (NoGo) in order to obtain rewards or avoid punishments. We replicated our previous finding that reward cues promoted actions nonselectively (Pavlovian response bias) while reward outcomes disproportionally facilitated credit assignment to selective actions (instrumental learning bias) [11]. Conversely, punishment cues inhibited actions nonselectively, whereas punishment outcomes reduced credit assignment following inaction, thereby facilitating sustained inaction. For half the cues, these motivational biases were congruent with the instrumental requirements, whereas the other cues required a response that was incongruent with the motivational biases (i.e., NoGo to gain reward and Go to avoid punishment). Oscillatory theta power increased over midfrontal sites for the motivationally incongruent cues when subjects correctly performed instrumental responses in which higher levels of midfrontal theta power were associated with reduced motivational biasing of action, in line with work by Cavanagh and colleagues [6]. Here, we assessed whether the reduced motivational biasing was driven by modulation of the Pavlovian response bias and/or the instrumental learning bias. To this end, we optimized the experimental paradigm to disentangle nonselective Pavlovian response tendencies from selective instrumental action values by including multiple active “Go” response options and showed that midfrontal theta power was associated with weaker contribution of the Pavlovian system (cf. M4a) rather than weaker instrumental learning biases (cf. M4d) or enhanced contribution of the instrumental system (cf. M4b–c). Finally, motor and lateral prefrontal sites synchronized to the midfrontal site in the theta frequency range, and these network dynamics explained the trial-by-trial reduction of the Pavlovian contribution over and above local, midfrontal theta power (cf. M5).

The midfrontal cortex has long been linked to performance monitoring and the detection of conflict, realizing the need for control [5,36–38] and increasing the expected value of control [39]. Detection of conflict has been suggested to be implemented as a coincidence detection in the midfrontal cortex, in which coactivation of competing response alternatives putatively generates theta-band oscillations [36]. In line with these accounts, we observed that oscillatory theta power over the midfrontal cortex covaried with the trial-by-trial conflict between Pavlovian and instrumental controllers, which might reflect coincidence detection within the midfrontal cortex of the coactivation of the competing Pavlovian and instrumental response tendencies. Moreover, midfrontal theta power seemed to be particularly modulated by Pavlovian–instrumental conflict when subjects were able to perform the correct instrumental response (see S7 Text), suggesting that subjects might not have detected conflict between the Pavlovian and instrumental controllers in the incorrect trials, leaving them to follow their prepotent, Pavlovian response tendencies. Using computational-model–based analyses, we showed that these midfrontal theta signals were particularly predictive of the trial-by-trial reduction of the Pavlovian response tendencies when Pavlovian and instrumental controllers conflicted (M4a). Note, however, that the midfrontal theta power did not seem to be a pure accuracy signal since midfrontal theta power was less predictive of the specific instrumental action values (cf. M4b), most closely reflecting the correct responses. Furthermore, we did not observe a link between midfrontal theta power and the instrumental learning bias, which taken together suggests that the midfrontal cortex is not responsible for unbiased, normative behavior per se but rather detects conflict between competing response systems. The midfrontal theta signal is indeed often considered to be uninformative about what the resulting response should be and instead would convey the signal that conflict resolution is needed [5,36]. In other words, midfrontal theta power might signal conflict, potentially allowing for an increase in the decision threshold [27–32,40] and thereby overcoming the Pavlovian response bias but without directly selecting an instrumental response. Although we link our work to previous studies demonstrating the relation between midfrontal theta power and the decision threshold (i.e., using drift diffusion models for two response options) [27,29,30], generalizing classic response conflict to Pavlovian–instrumental conflict, we could not directly test this relation in the current study, as these drift diffusion models are not suited to assess more than two response options.

The classical performance monitoring theory proposed that the need for control is signaled to the dorsolateral PFC, which will then implement the control [38]. More recent work suggested that the midfrontal cortex functions as a hub and signals the need for control to a wider network, including the lateral PFC, motor cortex, ventral striatum, and subthalamic nucleus, in order to increase the decision threshold [7,23–25,34,35]. Instead of assuming that the midfrontal cortex knows which areas to send the information to, the theta signal might be conveyed to all nodes in the network while only the active areas are susceptible for the midfrontal theta-band input [36], giving rise to activity-dependent functional connectivity. Accordingly, control-related functional connectivity has been observed between the midfrontal cortex and several target sites depending on the task at hand [5]. Consistent with these theories, we observed a motivational-conflict–related increase in phase synchrony between the midfrontal and motor sites and between the midfrontal and lateral prefrontal sites. Note, however, that techniques with higher spatial resolution (e.g., functional magnetic resonance imaging [fMRI]) are needed to determine whether we can attribute the lateral prefrontal synchronization to the dorsolateral PFC. Here, we reasoned that if control is instantiated by signaling the increased need for control to task-relevant sites [5,36], fluctuations in phase synchrony between the midfrontal and lateral prefrontal and motor sites would help to predict the reduction of the Pavlovian contribution. These network dynamics indeed explained the reduction of the Pavlovian response tendencies over and above local, midfrontal theta power. Thus, stronger trial-by-trial midfrontal phase synchrony with the lateral prefrontal and motor sites predicted reduced Pavlovian response biases better than midfrontal theta power alone. It should be noted that, although the estimation of theta phase angles is orthogonal to the estimation of theta power, these estimations are not completely independent, i.e., the phase estimation becomes more precise with more power. Yet, the ISPS explains behavioral performance over and above local theta power, whereas adding power to the synchrony models hardly improves model fit. Thus, the distal phase synchrony could account for the variance explained by local power while local power could not account for all variance explained by distal phase synchrony. Therefore, the synchrony results cannot purely be attributed to changes in power. Nevertheless, future studies could establish the contribution of network connectivity more independently by assessing connectivity measures that are independent of task-related local or network activation, such as structural or resting-state connectivity.

It has been proposed that the conflict-related intersite phase synchronization would have differential computational effects depending on the function of the target circuit; the lateral PFC might enhance the goal representations, whereas the motor cortex might increase the motor threshold [36]. Here, we did not observe evidence for these target-dependent effects. Specifically, we tested with alternative models whether synchronization of the lateral prefrontal sites was predictive of enhanced goal representations, i.e., instrumental action values, whereas synchronization of the motor sites was predictive of increased motor thresholds (S8 Text). These alternative models were inferior to models that implemented a direct modulation of the Pavlovian response bias, which might reflect the increased decision threshold enabling nonhardwired decision systems to take over. Altogether, these results seem to indicate that the theta-band network dynamics reflect the signaling of the need for control rather than signaling what specific computations are required within the target sites in order to implement the control. The resulting local computations could differ between target regions nonetheless but are not reflected in the theta-frequency band.

Finally, we have demonstrated increased midfrontal theta power for motivationally incongruent cues and linked trial-by-trial midfrontal theta power to the level of Pavlovian–instrumental conflict estimated from behavior. However, cognitive factors other than conflict might also play a role during motivational incongruency. Midfrontal theta power has been linked to the more general notion of “cognitive control” [5], including, e.g., conflict, novelty, punishment, error processing, and cognitive effort. While some factors (such as predicted response times and cue preferences) were orthogonal to motivational congruency in the current findings, we do not exclude the possibility that other factors (such as anxiety and cognitive effort allocation, which could be considered inherent to conflict resolution) might be important during motivational incongruency as well.

To summarize, in this study we replicate previous findings that i) the well-established motivational biasing of action arises partly from cue-based Pavlovian response biasing and partly from outcome-based instrumental learning biases [11] and that ii) oscillatory theta power over the midfrontal cortex is associated with reduced motivational biasing of action [6]. The midfrontal theta activity covaried with the level of conflict between Pavlovian and instrumental responses, putatively reflecting the detection of conflict, and was not linked to the instrumental learning biases. This conflict-related midfrontal signal was specifically associated with reduced prepotent, Pavlovian response tendencies, without selecting the specific instrumental response per se. Synchronization of the lateral prefrontal and motor sites to the midfrontal site predicted the reduced contribution of the Pavlovian system even better than the local, midfrontal activity, which highlights the importance of investigating distributed network processing in addition to local processing. Altogether, these findings suggest that the midfrontal cortex signals conflict to the network of task-related regions in order to putatively increase the decision threshold and thereby overcome the prepotent, Pavlovian responses and allow for goal-directed behavior.

## Methods

### Ethics statement

All procedures were approved by the local ethics committee (CMO/METC Arnhem Nijmegen: CMO2014/288) and performed in accordance with the Helsinki Declaration of 1975. Subjects signed informed consent prior to participation.

### Subjects

Thirty-four healthy adults participated in the study (aged 18–30 years, mean [SD] = 23.2 [3.6]; 27 females; right-handed; normal or corrected-to-normal vision). Exclusion criteria comprised a history of neurological or psychiatric disorders, use of psychotropic drugs, pregnancy, claustrophobia, and color blindness. EEG data of four subjects were excluded because of excessive muscle artifacts in the EEG signal (see EEG data acquisition and preprocessing). Subjects received a financial compensation or study credits upon completion of the experiment.

### Motivational Go/NoGo learning task

We employed a motivational Go/NoGo learning task with multiple active response options [11] to dissociate nonselective Pavlovian activation from biased instrumental learning of selective responses. In this learning task, subjects need to learn to make Go or NoGo responses to maximize rewards for “win” cues and minimize punishments for “avoid” cues (Fig 1).

Trials start with presentation of a gem-shaped cue (1,300 ms) followed by a fixation cross (700 ms) and feedback (1,000 ms). Four cues are followed by reward or neutral outcomes (“win” cues) and four cues by punishment or neutral outcomes (“avoid” cues). During cue presentation, subjects make a button press with the left hand (Go left) or right hand (Go right) or withhold from responding (NoGo). Only one of these response options is considered correct per cue, and based on the response, subjects receive feedback. Correct responses are followed by a reward (“win” cues) and a neutral outcome (“avoid” cues) 80% of the time and by a neutral outcome (“win” cues) and a punishment (“avoid” cues) otherwise. For incorrect responses, these probabilities are reversed. Based on the observed outcomes, subjects need to learn the optimal responses by trial and error. Trials end with an intertrial interval (ITI) varying from 1,000 ms to 1,750 ms in steps of 250 ms.

Subjects are informed that i) each cue can be followed by either reward or punishment, ii) each cue has one optimal response, iii) feedback is probabilistic, and iv) the rewards and punishments are converted to a monetary bonus upon completion of the study. The monetary bonus ranged from 0€–5€ (mean = 2.12, SD = 1.70). In contrast to our previous study [11], cue valence is not instructed and can be learned from the feedback. In total, there are eight cues with 40 trials per cue. After every 80 trials (approximately 6 min), subjects have a self-paced break. Each subject performed the task twice using two independent cue sets. The order of the cue sets is counterbalanced, and cue identities are randomized. The second round of the task is followed by a forced-choice transfer phase [6] (see S4 Text).

### EEG data acquisition and preprocessing

EEG data were acquired at 500 Hz from 65 channels (using a Brain Products actiCAP system; Brain Products, Gilching, Germany) placed according to the equidistant arrangement, under and above the left eye for vertical EOG and lateral to the eyes for horizontal EOG. The ground was placed on the forehead and the left mastoid was used for online referencing. EEG data are preprocessed and analyzed with the Fieldtrip software toolbox [41] in MATLAB (The MathWorks, Natick, MA, United States). EEG data were re-referenced offline to the weighted average of the mastoids, and the EEG signal of the reference electrode was recovered. Vertical and horizontal EOG electrodes were re-referenced into a bipolar montage. Data were high-pass filtered at 0.5 Hz and epoched into segments starting 1.75 s before cue onset and ending 1.5 s after feedback offset. The epochs were made sufficiently long to avoid edge artifacts resulting from time-frequency (TF) decomposition in the critical times of interest. Epochs were linear baseline corrected using the 200 ms prior to cue onset and visually inspected for trial rejection. Trials were rejected when containing EMG or artifacts unrelated to brain activity but not eye blinks. Four subjects had excessive EMG activity (>30% rejected trials) and were excluded from the EEG analyses. Of the remaining subjects, 1.7%–23.6% of the trials were rejected (mean = 10.5%). Independent component analysis (ICA) was performed over the EEG and vertical EOG data of the remaining epochs; components related to eye blinks or artifacts that were clearly distinguishable from brain activity were removed. One to five components were removed per subject (mean = 2.5). Two subjects had one channel containing flat lines; these channels were discarded and interpolated after ICA. Next, the EEG data were spatially filtered using estimation of the surface Laplacian [42,43]. The surface Laplacian filters out distant effects and accentuates local effects, thereby diminishing the effect of volume conduction on synchrony estimates [44].

### EEG TF decomposition

Cue- and response-locked time series were decomposed into their TF representations using wavelet convolution. Here, the time series are convolved with Morlet wavelets, i.e., sine waves convolved with a Gaussian model, based on multiplication in the frequency domain. The frequencies ranged from 1 Hz to 50 Hz in 39 logarithmically spaced steps, and the Gaussian width was fixed at four cycles. Wavelet convolution results in a complex signal from which power and phase are extracted for each TF point, down-sampled to 40 Hz. Condition-specific power values were averaged over trials and dB baseline corrected using a condition-averaged baseline condition (−250 ms to −50ms relative to cue onset). Phase angles (*φ*) were used to compute ISPS, which is thought to reflect intersite functional connectivity [24,45,46]. ISPS was computed for each TF point in the theta-frequency range (4 Hz–8 Hz) as follows: $ISPS\;=\;|\frac{1}{N}*\sum_{n\;=\;1}^{N}e^{i}\varphi_{j,t,f}-\varphi_{k,t,f}|$, where *N* is the number of trials, *i* the complex operator, and *j* and *k* the seed and target channels. ISPS values are sensitive to the number of trials, and therefore we selected the same number of trials from each condition to compute ISPS. This trial selection was permuted 100 times, and ISPS values were averaged over permutations. Cells with fewer than 20 trials were discarded for the analysis, resulting in the exclusion of two subjects for this analysis. ISPS values can range from 0 (no phase synchrony) to 1 (identical phase angles) and were baseline transformed into percent signal change using a condition-averaged baseline (again, −250 ms to −50 ms relative to cue onset).

For the single trial analyses, the Laplacian-filtered EEG data were broadband filtered in the theta range (4 Hz–8 Hz) and Hilbert transformed. Power time series were extracted, z transformed, averaged over the time window, and t-weighted for the channels of interest (Fig 3: channels 1 and 2; 450 ms–650 ms cue locked). The t-weighting was based on the main contrast (incongruent–congruent). Phase angles were extracted to compute phase synchrony between the midfrontal seed channels and motor and prefrontal target channels during the same time window: $ISPS\;=\;|\frac{1}{t}*\sum_{t\;=\;1}^{T}e^{i}\varphi_{j,n}-\varphi_{k,n}|$. Here, the seed channels were weighted identical to the power time series, and the target channels were t-weighted by the corresponding main ISPS contrast (lateral prefrontal: valence × required action; motor channels: congruency × motor Execution; Fig 5). The resulting power and ISPS values were inverse transformed before use in the computational models.

### EEG channel selection

**Fig 6:**
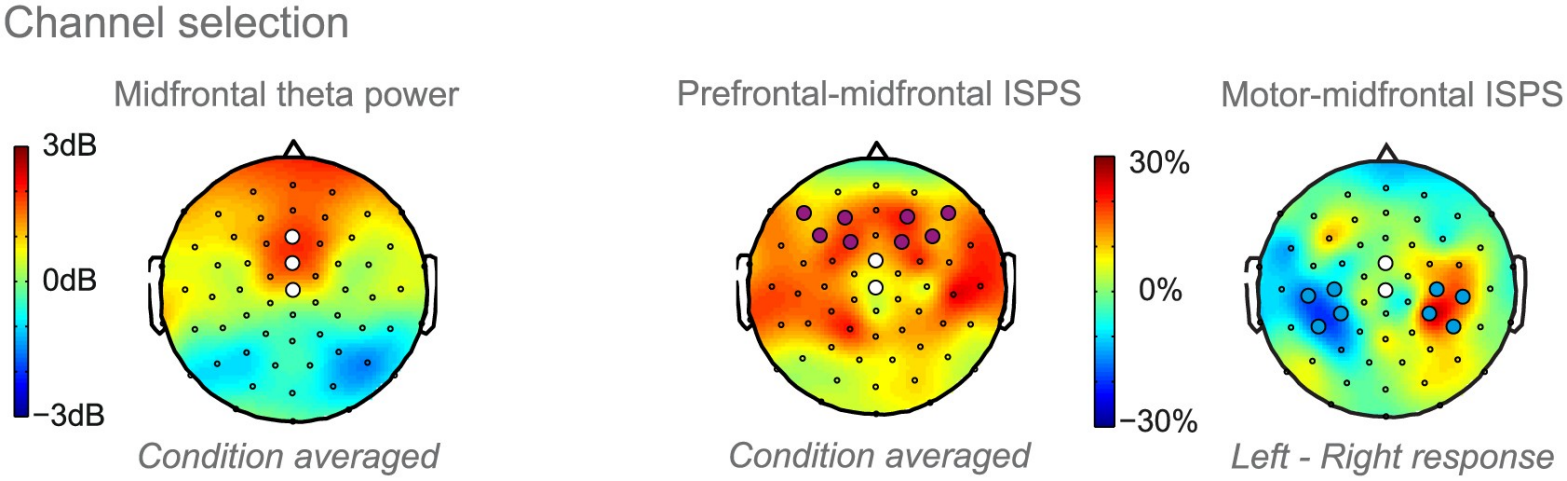
Channel selection. Left: Topographic distribution of condition-averaged theta power (4 Hz–8 Hz). The three white discs indicate the midfrontal cluster. Right: Topographic distribution of ISPS with the midfrontal channels serving as t-weighted seed. Only the two channels showing the effect of interest (see Fig 3) were included as seed channels. The lateral prefrontal target channels (purple discs) were selected based on the strongest condition-averaged midfrontal phase synchrony and extended to include all channels surrounding F5/6 in the standard 10–20 montage, given that F5/6 are commonly assessed as dorsolateral prefrontal channels; the motor target channels (blue discs) were selected based on response lateralization (i.e., left–right responses). Underlying data can be found at http://hdl.handle.net/11633/di.dccn.DSC_3017033.03_624. ISPS, intersite phase synchrony.

For all analyses, we selected clusters of channels informed by prior work and fine-tuned the clusters in a data-driven manner (independent of the contrasts of interest), as scalp topographies can vary considerably between subjects and studies. Note that we recorded EEG with an equidistant channel arrangement instead of the more commonly used 10–20 arrangement. To assess midfrontal theta power, we selected two central channels corresponding to Cz and FCz in the 10–20 arrangement, as these channels showed a robust modulation of midfrontal theta power to response conflict in previous work [24,47]. We then fine-tuned this midfrontal cluster based on the condition-averaged cue-locked data (orthogonal to the contrast of interest) by including one additional anterior channel, roughly corresponding to Fz (see Fig 6). With post hoc *t* tests, we assessed which of the channels contributed significantly to significant group-level cluster effects (Bonferroni corrected alpha = 0.017). We then combined these post hoc significant channels using t-weighting based on the main contrast (incongruent–congruent; also see the Statistical analysis section) and entered the t-weighted trial-wise data in the computational models. To emphasize, the t-weighting was only performed after establishing the effect of interest at the group level and thus did not bias our results. We performed the t-weighting at the group level rather than the subject level to reduce proneness to noise and enhance generalizability. To assess phase synchronization of the lateral PFC and motor cortex to the midfrontal cortex, we computed a t-weighted seed time series based on the significant midfrontal channels. We selected the following target clusters: i) eight dorsolateral prefrontal channels, including the dorsolateral peaks observed in the condition averaged data and extended laterally to surround the channels F5/6 in the 10–20 EEG montage (traditionally considered dorsolateral prefrontal channels [18,23,48]); and ii) four left and four right motor channels, for which we observed a clear lateralization based on the response hand independent of cue valence. See Fig 6 for the channel selection.

### Statistical analysis

The behavioral data were analyzed in line with Swart and colleagues [11]. We elaborate on the details of the statistical analyses and the computational models in S1 and S2 Text, respectively.

To assess whether midfrontal theta power is related to reduced Pavlovian response biases, we analyzed TF power for the midfrontal cluster and restricted the analysis to the frequency range of interest (4 Hz–8Hz) and to the response window, i.e., cue presentation. We employed a time-based permutation test (500 permutations) with the cue-locked, trial-averaged midfrontal theta power as dependent variable and the within-subject factor congruency (congruent versus incongruent); the Go-to-Win and NoGo-to-Avoid cues are considered to be motivationally congruent, as the Pavlovian response tendencies are in line with the instrumental requirements, whereas the NoGo-to-Win and Go-to-Avoid cues are considered to be motivationally incongruent. With post hoc *t* tests, we assessed which of the three midfrontal channels contributed significantly to the resulting time window (Bonferroni-corrected alpha = 0.017). Given that i) midfrontal theta power is known to strongly increase after errors [34,49] and ii) errors are more prevalent on motivationally incongruent than congruent trials, we assessed correct trials only to minimize the influence of error processing on the midfrontal theta signal. We report the analysis of incorrect trials in S7 Text. We repeated the analysis for response-locked power (Go cues only).

To test whether midfrontal theta power might also be related to reduced instrumental learning biases, we assessed feedback-related midfrontal theta power, which has been linked to reinforcement learning [5,18]. In parallel to the cue-locked analysis, we selected the same midfrontal cluster and frequency range (4 Hz–8 Hz) and restricted the analysis to the period of feedback presentation. To retrieve the time window related to feedback processing, we first employed a time-based permutation test (500 permutations) with the feedback-locked, trial-averaged midfrontal theta power as a dependent variable and the within-subject factor outcome (preferred versus nonpreferred); reward following “win” cues and neutral feedback following “avoid” cues are considered to be preferred, whereas neutral feedback following “win” cues and punishment following “avoid” cues are considered to be nonpreferred. Thus, the time window resulting from this permutation test differentiated between the feedback. We then extracted power for the resulting TF window and contrasted the conditions with biased learning (enhanced learning for rewarded Go responses and decreased learning for punished NoGo responses) to their unbiased counterparts (rewarded NoGo and punished Go). Note that we only provide statistics for effects orthogonal to the main effect of outcome, given that we selected the time window based on the outcome contrast, rendering statistics for this contrast invalid.

To assess phase synchronization of the lateral PFC and motor cortex to the midfrontal cortex, we assessed the t-weighted phase synchronization between the midfrontal cluster and the lateral prefrontal and motor clusters. We extracted the ISPS values for the significant time window of the cue-locked permutation test using correct trials. We analyzed ISPS using a repeated measures ANOVA with the factors valence (“win” versus “avoid” cue) × required action (Go versus NoGo) to assess whether ISPS increased during motivationally incongruent cues. For the motor channels, we reasoned that ISPS might be differentially affected in the “executing” motor cortex (i.e., contralateral to the Go response hand) relative to the “nonexecuting” motor cortex (i.e., ipsilateral to the Go response hand). To elaborate, midfrontal functional connectivity is thought to be activity dependent [36] such that target sites become more susceptible to the midfrontal signals when they are more active. The motor cortex putatively becomes more activated during contralateral Go responses than during ipsilateral or NoGo responses. To this end, we split up the Go responses into contra- and ipsilateral, resulting in three levels for the factor required response (Go_contra_/Go_ipsi_/NoGo_bilateral_). For the significant valence × required response interaction, we then assessed whether this interaction could be explained by an effect of motivational congruency in the nonexecuting motor channels (ipsilateral Go and bilateral NoGo responses) versus executing motor channels (contralateral Go).

## Supporting information

S1 Text Statistical analysis of behavioral data. (DOCX)

S2 Text Computational models. (DOCX)

S3 Text Reaction times. (DOCX)

S4 Text Forced-choice transfer phase. (DOCX)

S5 Text Feedback-related midfrontal theta power. (DOCX)

S6 Text Midfrontal–motor phase synchrony. (DOCX)

S7 Text Single trial power and ISPS. ISPS, intersite phase synchrony.
(DOCX)

S8 Text Computational modeling: Potential alternative mechanisms related to ISPS. ISPS, intersite phase synchrony.
(DOCX)

S1 Table Median (25th–75th percentile) of subject-level parameter estimates in model space. (XLSX)

S2 Table Confidence of group-level parameter estimates (% of samples > 0). (XLSX)

## Abbreviations

EEG: electroencephalography
EMG: electromyography
EOG: electro-oculography
fMRI: functional magnetic resonance imaging
ICA: independent component analysis
ISPS: intersite phase synchrony
ITI: intertrial interval
PFC: prefrontal cortex
TF: time frequency
WAIC: Watanabe–Akaike Information Criterion
