# Supplementary material for: Frontal network dynamics reflect neurocomputational mechanisms for reducing maladaptive biases in motivated action
Source: PLoS Biol. 2018 Oct 18;16(10):e2005979. doi: 10.1371/journal.pbio.2005979 (PMC6207318; doi:10.1371/journal.pbio.2005979)
Supplement: S1 Text — (DOCX) [file pbio.2005979.s001.docx]

**S1 Text. Statistical analysis of behavioral data.**

Behavioral data were analyzed in line with Swart et al. (2017). In short, to assess the influence of motivational valence on behavioral activation, we first analyzed Go vs. NoGo responses (irrespective of Go-left vs. Go-right) as a function of cue valence. Second, we tested whether motivational valence affected both correct and incorrect Go responses. To account for both between and within subject variability, choice data were analyzed with logistic mixed-level models using the lme4 package in R[2,3]. These mixed models included the within subject factors Valence (Win vs. Avoid cue) and Required Action (Go vs. NoGo). The analysis of correct and incorrect Go responses included only Go cues, and hence only the factor Valence. Models included all main effects and interactions, and a full random effects structure[4,5]. For completeness, we analyzed reaction times (RTs) as a measure of behavioral vigor (see S3 Text).

**References**

1. Swart JC, Froböse MI, Cook JL, Geurts DEM, Frank MJ, Cools R, et al. Catecholaminergic challenge uncovers distinct Pavlovian and instrumental mechanisms of motivated (in)action. Elife. 2017;6. doi:10.7554/eLife.22169

2. Bates D, Maechler M, Bolker B, Walker S. lme4: Linear mixed-effects models using Eigen and S4. R package version 1.1-7, http://CRAN.R-project.org/package=lme4. R Packag version. 2014; doi:citeulike-article-id:7112638

3. R Developement Core Team. R: A Language and Environment for Statistical Computing. R Found Stat Comput. 2015;1: 409. doi:10.1007/978-3-540-74686-7

4. Barr DJ, Levy R, Scheepers C, Tily HJ. Random effects structure for confirmatory hypothesis testing: Keep it maximal. J Mem Lang. 2013;68: 255–278. doi:10.1016/j.jml.2012.11.001

5. Barr DJ. Random effects structure for testing interactions in linear mixed-effects models. Front Psychol. 2013;4: 328. doi:10.3389/fpsyg.2013.00328
