# Supplementary material for: Frontal network dynamics reflect neurocomputational mechanisms for reducing maladaptive biases in motivated action
Source: PLoS Biol. 2018 Oct 18;16(10):e2005979. doi: 10.1371/journal.pbio.2005979 (PMC6207318; doi:10.1371/journal.pbio.2005979)
Supplement: S2 Text — (DOCX) [file pbio.2005979.s002.docx]

**S2 Text. Computational models.**

First, we set out to replicate the finding that the Pavlovian response bias and instrumental learning bias contribute to the well-established asymmetric effects of valence on behavioral activation. To this end we employ the same computational modeling approach as previously[1]. Using nested reinforcement learning models, we formally test whether i) *cue* valence biases behavioral activation in a Pavlovian manner, such that Win cues promote non-selective Go responses and Avoid cues promote NoGo responses, ii) *outcome* valence biases instrumental learning of (in)action; reward outcomes are more potent in reinforcing active actions, whereas punishment outcomes are less potent in driving unlearning of holding back. Next, we extend the winning model with trial-by-trial estimates of theta power (model family M4), to assess whether these trial-by-trial neural measures increase our ability to explain behavioral responses. In particular, we assess whether on incongruent trials theta power is predictive of a) reduced Pavlovian response biases, b) enhanced impact of instrumental values, c) altered balance between these two systems, or d) reduced instrumental learning biases. Finally, we assess for the winning model, whether replacing local theta power with intersite phase synchrony in the theta range with putative target structures (lateral prefrontal cortex, motor cortex) further explains trial-by-trial behavioral responses (model family M5). Below we will describe the details of the models.

In all models, the probability of each response (*a*) is estimated based on computed action weights (*w*) using a softmax function:

|  | $p\left( a_{t} \vert s_{t} \right)= \left[ \frac{exp(w(a_{t},s_{t}))}{\sum_{a'} exp(w(a',s_{t}))} \right]$ | Eq. 1 |
| --- | --- | --- |

Where *t* indicates the trial and *s* the state (i.e. the cue). In the simplest model (M1) the action weights are fully determined by the learned values of each action (Q-values). Action values are updated with the Q-value based prediction error, i.e. the deviation of the observed outcome from the expected outcome (standard delta-rule learning[2], see Eq. 2). M1 contains two free parameters: a learning rate (ε) scaling the prediction-error, and feedback sensitivity (ρ) scaling the outcome value:

|  | $Q_{t}\left( a_{t},s_{t} \right)= Q_{t-1}\left( a_{t},s_{t} \right)+ \varepsilon\left( {\rho r}_{t}- Q_{t-1}\left( a_{t},s_{t} \right) \right)$ | Eq. 2 |
| --- | --- | --- |

In Eq. 2, outcomes are reflected by *r,* where r *∈*{-1,0,1}. As subjects can infer the cue valence upon the first reward or punishment outcome, neutral outcomes can only be evaluated as optimal (Avoid cues) or suboptimal (Win cues) after the first reward or punishment outcome. Accordingly, we tested whether subjects revalue previously experienced neutral outcomes as (sub)optimal upon the first reward or punishment. To this end, initial *Q*-values (*Q*_0_) are set to *ρ**0.5 for Win cues and *ρ**-0.5 for Avoid cues (i.e. the initial expected outcome is 0.5 for Win cues and -0.5 for Avoid cues), whereas these Q-values affect behavior only once the cue valence is known (Eq. 3). Thus, as an example, if a subject made 5 NoGo responses to a cue and receives 5 neutral outcomes, the subject will learn that NoGo leads to a neutral outcome, but, not knowing whether this is a ‘Win’ or ‘Avoid’ cue, does not know whether they are aiming to get a neutral outcome. If on the 6^th^ trial the NoGo response is followed by a punishment or reward (because of the probabilistic feedback), the subject can suddenly deduce that the NoGo response was actually optimal (in case of punishment) or suboptimal (in case of reward), given that they know by instruction that cues can only lead to reward or punishment. Modelling this revaluation increases model evidence relative to model M1 without revaluation of neutral outcomes (ΔWAIC=-37). Note that this revaluation adds a model-based component to our otherwise overall model-free learning system (cf. Eq.2).

$q\left( a_{t},s_{t} \right)= \left\{ \begin{matrix} Q\left( a_{t},s_{t} \right) if \{\left| r_{1\ldots t} \right|\} \ni1 \\ 0 else \end{matrix} \right.$ Eq. 3

In M2, a Go bias parameter (*b*) was added to the action weights of Go responses to account for individual differences in the tendency to make Go responses independent of any cue. M3a then adds the influence of a Pavlovian response bias (π), where static Pavlovian values (*V*) contribute to the action weights:

|  | $w\left( a_{t},s_{t} \right)= \left\{ \begin{matrix} q\left( a_{t},s_{t} \right)+ \pi V\left( s \right)+ b if a=Go \\ q\left( a_{t},s_{t} \right) else \end{matrix} \right.$ | Eq. 4 |
| --- | --- | --- |

Here V=0.5 for Win cues, and V=-0.5 for Avoid cues. In this model, positive Pavlovian values increase the action weight of all Go responses, where π scales the weight of the Pavlovian values. As the cue valence can only be inferred upon the first reward or punishment, V(s)=0 beforehand. Alternatively, Pavlovian values might have been learned using delta-rule learning[3]: $V_{t}\left( s_{t} \right)= V_{t-1}\left( s_{t} \right)+ \varepsilon\left( r_{t}- V_{t-1}\left( s_{t} \right) \right)$. However, prediction-error based learning of Pavlovian values greatly reduced model evidence (ΔWAIC=+323), and thus we proceeded with a static Pavlovian influence.

In M3b, we included an instrumental learning bias parameter (κ), to assess whether reward is more effective in reinforcing Go responses than NoGo responses, whereas punishment is less effective in unlearning NoGo responses than Go responses. In this model, κ increases the learning rate for rewarded Go responses and decreases the learning rate for punished NoGo responses:

|  | $\varepsilon= \left\{ \begin{matrix} \varepsilon_{0}+\kappa if r_{t}= 1 \& a=go \\ \varepsilon_{0}-\kappa if r_{t}=-1 \& a=nogo \\ \varepsilon_{0} else \end{matrix} \right.$ | Eq. 5 |
| --- | --- | --- |

M3c included both the π and κ parameter to test whether the Pavlovian response bias and instrumental learning bias complementarily contribute to the observed motivational bias in action.

In the winning behavioral model (M3c), we tested whether midfrontal theta power covaried with the level of Pavlovian-instrumental conflict, which would be in line with the hypothesis that the midfrontal cortex is involved in the detection of motivational conflict. We computed the level of conflict as the difference in Q-values for the Pavlovian congruent versus incongruent responses. Thus, motivational conflict was computed as Q_nogo_ - mean(Q_go_) for Win cues, and as mean(Q_go_) - Q_nogo_ for Avoid cues.

After establishing the contribution of the cue- and outcome-based motivational biases, we continued our computational modelling approach to assess the functional role of cue-locked midfrontal theta power in overcoming the motivational biases. We used competing models (M4a-d; cf. Cavanagh et al., 2013) to assess the potential mechanisms by which the midfrontal cortex might help to overcome the motivational biases. In these models, we quantified the impact of trial-by-trial estimates of midfrontal theta power ($\theta_{t}$). The extended EEG models were estimated for all subjects that were included in the EEG analyses (i.e. excluding 4 subjects). To this end, we re-estimated the winning base model over the 30 EEG subjects, which left parameter inference unaffected, and used the resulting model evidence for model comparison in the EEG section.

In model M4a we tested whether midfrontal theta power relates to modulation of the impact of the Pavlovian bias on behavior:

|  | $w\left( {Go'}_{t},s_{t} \right)= \left\{ \begin{matrix} Q\left( {Go'}_{t},s_{t} \right)+\left( \pi+ \beta*\theta_{t} \right)*V\left( s \right)+ b if conflict \\ Q\left( {Go'}_{t},s_{t} \right)+ \pi V\left( s \right)+ b else \end{matrix} \right.$ | Eq. 6 |
| --- | --- | --- |

Here, the β parameter scales the impact of the midfrontal theta estimates on the Pavlovian response tendencies. Positive β estimates indicate that midfrontal theta power enhances the Pavlovian response tendencies, while negative β estimates reduce the Pavlovian response tendencies. Theta power only scales the impact of the Pavlovian bias on conflict trials (NoGo-to-Win, Go-to-Avoid), in line with previous report[4].

In M4b we tested whether midfrontal theta power relates to modulation of the instrumental contribution. Here, the β parameter allows midfrontal theta estimates to scale the impact of the instrumental Q-values on the action weights:

$w\left( {Go'}_{t},s_{t} \right)= \left\{ \begin{matrix} \left( 1- \beta*\theta_{t} \right)*Q\left( {Go'}_{t},s_{t} \right)+\pi V\left( s \right)+ b if conflict \\ Q\left( {Go'}_{t},s_{t} \right)+ \pi V\left( s \right)+ b else \end{matrix} \right.$ Eq. 7

$w\left( {NoGo}_{t},s_{t} \right)= \left\{ \begin{matrix} \left( 1- \beta*\theta_{t} \right)*Q\left( {NoGo}_{t},s_{t} \right) if conflict \\ Q\left( {NoGo}_{t},s_{t} \right) else \end{matrix} \right.$

In M4c we tested whether midfrontal theta power relates to modulation of the relative balance between the Pavlovian and instrumental control systems, using a Pavlovian-instrumental trade-off parameter τ instead of the Pavlovian bias parameter π. The β parameter allows midfrontal theta estimates to shift the balance between Pavlovian versus instrumental control:

$w\left( {Go'}_{t},s_{t} \right)= \left\{ \begin{matrix} \left( 1-(\tau+ \beta*\theta_{t} \right))*Q\left( {Go'}_{t},s_{t} \right)+\left( \tau+ \beta*\theta_{t} \right)*V\left( s \right)+ b if conflict \\ \left( 1-\tau\right)*Q\left( {Go^{'}}_{t},s_{t} \right)+ \tau*V\left( s \right)+ b else \end{matrix} \right.$ Eq. 8

$w\left( {NoGo}_{t},s_{t} \right)= \left\{ \begin{matrix} \left( 1-(\tau+ \beta*\theta_{t} \right))*Q\left( {NoGo}_{t},s_{t} \right) if conflict \\ \left( 1-\tau\right)*Q\left( {NoGo}_{t},s_{t} \right) else \end{matrix} \right.$

The models M4a-c assess the mechanisms proposed by Cavanagh et al. (2013), and here we can optimally disentangle the Pavlovian and instrumental control systems due to the multiple Go response options. Furthermore, in our previous work we established that motivational biases arise not only from cue-based Pavlovian mechanisms, but also from biased instrumental learning. We replicated this finding here showing the superiority of model M3c. This suggests that there is also a fourth mechanism by which midfrontal cortical control may help to respond correctly when Pavlovian and instrumental controllers conflict, namely that midfrontal theta power relates to reducing the instrumental learning bias. We test this hypothesis in model M4d, by allowing mid-frontal theta power to scale the motivational bias in learning rates:

|  | $\varepsilon_{rewarded Go}= \left\{ \begin{matrix} \left( 1-\beta*\theta_{t} \right)*{(\varepsilon}_{0}+\kappa)+(\beta*\theta_{t})*\varepsilon_{0} if conflict \\ \varepsilon_{0}+\kappa else \end{matrix} \right.$ | Eq. 9 |
| --- | --- | --- |

$$\varepsilon_{punished NoGo}= \left\{ \begin{matrix} \left( 1-\beta*\theta_{t} \right)*{(\varepsilon}_{0}-\kappa)+(\beta*\theta_{t})*\varepsilon_{0} if conflict \\ \varepsilon_{0}-\kappa else \end{matrix} \right.$$

In contrast to M4a-c, here the $\beta$ parameter was [0 1] constrained, such that the biased learning rates ($\varepsilon_{rewarded Go}$ and $\varepsilon_{punished NoGo}$) could regress towards the unbiased learning rate ($\varepsilon_{0}$), ensuring that the resulting learning rates could not go out of [0 1] bounds (see parameter constraints below). Accordingly, a positive $\beta$ estimate indicates midfrontal theta power reduces the instrumental learning bias, rendering instrumental learning more unbiased.

Having established the computational mechanism through which midfrontal theta may reduce motivational biases (winning model M5a), we assessed whether this control might be instantiated by synchronization of the task-relevant regions to the midfrontal cortex. Current theories suggest that one role of the midfrontal cortex is to detect motivational conflict and ‘alert’ task-relevant regions to implement this control, through synchronization of the task-relevant regions to the midfrontal cortex[5,6]. These ideas suggest that perhaps the degree of synchronization with task-relevant regions, rather than local power, would be better predictors of the ability to reduce motivational biases. To this end, we assessed whether theta phase synchronization between midfrontal channels and the bilateral prefrontal channels (ISPS_PFC_, M5a) or the contralateral motor channels (ISPS_motor-contra_, M5b) scaled the Pavlovian bias, and explained behavior better than midfrontal theta power. Accordingly, we replaced $\theta_{t}$ in Eq. 6 with trial-by-trial phase synchrony in the theta band of midfrontal to lateral prefrontal and contralateral motor channels.

We used a sampling method for hierarchical Bayesian estimation of group-level and subject-level parameters. The group-level parameters (*X*) serve as priors for the individual-level parameters (*x*), such that *x* ~ *Ɲ*(*X*,*σ*). The hyperpriors for *σ* are specified by a half-Cauchy[7] with a scale of 2. The hyperpriors for *X* are centered around 0 and weakly informative: *X_ε,κ_* ~ *Ɲ*(0,2), *X_ρ,b,π,β_* ~ *Ɲ*(0,3). All parameters are unconstrained, with the exception of *ρ* (positivity constraint implemented using an exponential transform), ε ([0 1] constraint implemented with an inverse logit transform), and $\beta$ in M5d ([0 1] constraint; i.e. $\beta$ could suppress, but not reverse, the learning bias). To ensure that the effect of κ on ε (Eq.5) was symmetrical in model space (i.e. after inverse logit transformation to ensure [0 1] constraint), ε was computed as:

|  | $\varepsilon= \left\{ \begin{matrix} \varepsilon_{0}=inv.logit\left( \varepsilon\right) \\ \varepsilon_{punished NoGo}=inv.logit\left( \varepsilon-\kappa\right) if \varepsilon_{0}< .5 \\ \varepsilon_{rewarded Go}= \varepsilon_{0}+\left( \varepsilon_{0}-\varepsilon_{punished NoGo} \right) if \varepsilon_{0}< .5 \end{matrix} \right.$ | Eq. 10 |
| --- | --- | --- |

$\varepsilon= \left\{ \begin{matrix} \\ \varepsilon_{rewarded Go}= inv.logit\left( \varepsilon+\kappa\right) if \varepsilon_{0}> .5 \\ \varepsilon_{punished NoGo}=\varepsilon_{0}+\left( \varepsilon_{0}-\varepsilon_{rewarded Go} \right) if \varepsilon_{0}> .5 \end{matrix} \right.$

Model estimation was performed using Stan software in R (RStan)[8]. Stan provides full Bayesian inference with Markov chain Monte Carlo (MCMC) sampling methods[9]. The number of Markov chains was set at 4, with 200 burn-in iterations and 1000 post burn-in iterations per chains (4000 total). Model convergence was considered when the potential scale reduction factor $\hat{R}$<1.1 for all parameters[10], and all models reached convergence accordingly. Model comparison was evaluated using the Watanabe-Akaike Information Criteria (WAIC)[11]. WAIC is an estimate of the likelihood of the data given the model parameters, penalized for the effective number of parameters to adjust for overfitting. Lower (i.e. more negative) WAIC values indicate better model fit. As WAIC is reported on the deviance scale[12], a difference in WAIC value of 2–6 is considered positive evidence, 6–10 strong evidence, and >10 very strong evidence[13]. We additionally provide a measure of explained variance (R^2^) for the models, as R^2^ might be considered more intuitive. However, WAIC is the most appropriate measure to compare models as WAIC penalizes for increasing model complexity. Moreover, WAIC takes into account how much variance a parameter could explain (for example, while the Pavlovian bias impacts all trials, the EEG model parameters only have an impact on the incongruent trials and can thereby explain less variance). In contrast, the R^2^ values do not account for the number of parameters and the extent to which a parameter is restricted in explaining variance.

**References**

1. Swart JC, Froböse MI, Cook JL, Geurts DEM, Frank MJ, Cools R, et al. Catecholaminergic challenge uncovers distinct Pavlovian and instrumental mechanisms of motivated (in)action. Elife. 2017;6. doi:10.7554/eLife.22169

2. Sutton RS, Barto AG. Reinforcement Learning: An Introduction. IEEE Trans Neural Networks. 1998;9: 1054–1054. doi:10.1109/TNN.1998.712192

3. Guitart-Masip M, Huys QJM, Fuentemilla L, Dayan P, Duzel E, Dolan RJ. Go and no-go learning in reward and punishment: Interactions between affect and effect. Neuroimage. 2012;62: 154–166. doi:10.1016/j.neuroimage.2012.04.024

4. Cavanagh JF, Eisenberg I, Guitart-Masip M, Huys Q, Frank MJ. Frontal Theta Overrides Pavlovian Learning Biases. J Neurosci. 2013;33: 8541–8548. doi:10.1523/JNEUROSCI.5754-12.2013

5. Cohen MX, Cavanagh JF. Single-trial regression elucidates the role of prefrontal theta oscillations in response conflict. Front Psychol. 2011;2. doi:10.3389/fpsyg.2011.00030

6. Cavanagh JF, Frank MJ. Frontal theta as a mechanism for cognitive control. Trends in Cognitive Sciences. 2014. pp. 414–421. doi:10.1016/j.tics.2014.04.012

7. Gelman A. Prior distribution for variance parameters in hierarchical models. Bayesian Anal. 2006;1: 515–533. doi:10.1214/06-BA117A

8. Stan Development Team. RStan: the R interface to Stan, Version 2.9.0. [Internet]. 2016. Available: http://mc-stan.org

9. Metropolis N, Rosenbluth AW, Rosenbluth MN, Teller AH, Teller E. Equation of state calculations by fast computing machines. J Chem Phys. 1953;21: 1087–1092. doi:http://dx.doi.org/10.1063/1.1699114

10. Gelman A, Rubin DB. Inference from Iterative Simulation Using Multiple Sequences. Statistical Science. 1992. pp. 457–511. doi:10.1214/ss/1177011136

11. Watanabe S. Asymptotic Equivalence of Bayes Cross Validation and Widely Applicable Information Criterion in Singular Learning Theory. J Mach Learn Res. 2010;11: 3571–3594. Available: http://www.jmlr.org/papers/volume11/watanabe10a/watanabe10a.pdf

12. Gelman A, Hwang J, Vehtari A. Understanding predictive information criteria for Bayesian models. Stat Comput. 2014;24: 997–1016. doi:10.1007/s11222-013-9416-2

13. Kass R, Raftery A. Bayes Factors. J Am Stat Assoc. 1995;90: 773–795. doi:10.1080/01621459.1995.10476572
