# Supplementary material for: Frontal network dynamics reflect neurocomputational mechanisms for reducing maladaptive biases in motivated action
Source: PLoS Biol. 2018 Oct 18;16(10):e2005979. doi: 10.1371/journal.pbio.2005979 (PMC6207318; doi:10.1371/journal.pbio.2005979)
Supplement: S3 Text — (DOCX) [file pbio.2005979.s003.docx]

**S3 Text. Reaction times.**

For completeness, we analyzed reaction times (RTs) as a measure of behavioral vigor. RTs were analyzed with linear mixed-level models using the lme4 package in R[1,2]. First, we assessed RTs irrespective of accuracy, with a mixed model including the within subject factors Valence (Win vs. Avoid cue) and Required Action (Go vs. NoGo). Second, we assessed RTs for the Go cues as a function of accuracy. This mixed model included the within subject factors Valence (Win vs. Avoid cue) and Accuracy (correct vs. incorrect). RTs were ln-transformed to improve normality. Models included all main effects and interactions, and a full random effects structure[3,4].

The response times echoed the effects on proportion of Go responses. Learning was evidenced by shorter RTs for Go than NoGo cues (X^2^(1)=62.3, *p*<.001) and for correct relative to incorrect Go responses (X^2^(1)=88.3, *p*<.001). Motivational biasing was also evident from RTs, as subjects responded faster to Win vs. Avoid cues (X^2^(1)=98.6, *p*<.001) independent of the response requirements (X^2^(1)<1, *p*=.979) or accuracy (X^2^(1)=1.7, *p*=.198). Importantly, the effect of cue valence on RTs covaried with the effect on proportion of Go responses, such that subjects with a higher proportion of Go responses to Win cues also sped up more for Win cues (R_pearson_=-.53, *p*=.001), suggesting that the same neural mechanisms underlying the motivational biases drove changes in both RT and choice.

**References**

1. Bates D, Maechler M, Bolker B, Walker S. lme4: Linear mixed-effects models using Eigen and S4. R package version 1.1-7, http://CRAN.R-project.org/package=lme4. R Packag version. 2014; doi:citeulike-article-id:7112638

2. R Developement Core Team. R: A Language and Environment for Statistical Computing. R Found Stat Comput. 2015;1: 409. doi:10.1007/978-3-540-74686-7

3. Barr DJ, Levy R, Scheepers C, Tily HJ. Random effects structure for confirmatory hypothesis testing: Keep it maximal. J Mem Lang. 2013;68: 255–278. doi:10.1016/j.jml.2012.11.001

4. Barr DJ. Random effects structure for testing interactions in linear mixed-effects models. Front Psychol. 2013;4: 328. doi:10.3389/fpsyg.2013.00328
