# Supplementary material for: Frontal network dynamics reflect neurocomputational mechanisms for reducing maladaptive biases in motivated action
Source: PLoS Biol. 2018 Oct 18;16(10):e2005979. doi: 10.1371/journal.pbio.2005979 (PMC6207318; doi:10.1371/journal.pbio.2005979)
Supplement: S4 Text — (DOCX) [file pbio.2005979.s004.docx]

**S4 Text. Forced-choice transfer phase.**

At the end of the learning task, subjects performed a forced choice transfer phase. In the transfer phase, cues from the last round appear on screen in pairs; subjects are requested to select the cue they found most rewarding. These explicit relative preferences provide a measure of the learned cue values. No feedback is presented at this stage, minimizing interference with the learned cue values. Cues are presented above and below the center of the screen to be orthogonal to the left and right response requirements of the learning phase. All possible cue pairs are presented twice, with counterbalanced location, except for the pairs with cues from the same category (i.e. Go-to-Win/Go-to-Avoid/NoGo-to-Win/NoGo-to-Avoid). The transfer phase contained 48 trials in total.

All subjects indicated the Win cues as rewarding more often than the Avoid cues (*t*_33_=36.5, *p*<.001), confirming that subjects learned the cue values. On top of that, subjects also preferentially indicated the Go cues over the NoGo cues as more rewarding (*t*_33_=3.1, *p*=.004), even though the received outcomes did not differ significantly (*t*_33_=1.7, *p*=.106). Thus, the preference of Go cues did not seem to reflect higher outcomes associated with these cues per se. In other words, the subjective values were boosted for Go vs. NoGo cues, which could not solely be explained by differential outcomes.
