# Supplementary material for: Frontal network dynamics reflect neurocomputational mechanisms for reducing maladaptive biases in motivated action
Source: PLoS Biol. 2018 Oct 18;16(10):e2005979. doi: 10.1371/journal.pbio.2005979 (PMC6207318; doi:10.1371/journal.pbio.2005979)
Supplement: S5 Text — (DOCX) [file pbio.2005979.s005.docx]

**S5 Text. Feedback-related midfrontal theta power.**

In the main text, we addressed modulations of feedback-related midfrontal theta power as a function of the biased instrumental learning (i.e. enhanced Go learning after reward and hampered NoGo unlearning after punishment). Here, we report a more extensive analysis of feedback-related power using a repeated measures ANOVA with the Factors Outcome (Preferred vs. Non-preferred) x Valence (Win vs. Avoid cue) x Required Response (Go vs. NoGo). Independent of the biased learning conditions, we observed clear effects of cue valence, such that feedback-related midfrontal theta power increased for Avoid relative to Win cues (*F*_1,29_=35.6, *p*<.001). Additionally, the outcome effect (non-preferred vs. preferred outcomes) was stronger for the Avoid cues (Avoid cues: *F*_1,28_=24.2, *p*<.001; Outcome x Valence: *F*_1,28_=4.6, *p*=.042), though also highly significant for the Win cues (Win cues: *F*_1,28_=11.9, *p*=.002). Interestingly, midfrontal theta power did not significantly differ for neutral outcomes following a Win vs. Avoid cue (*F*_1,28_=1.8, *p*=.189), despite their relative difference (i.e. non-preferred vs. preferred outcome). Thus, we observed well-established feedback-related modulations of midfrontal theta power[1], yet this feedback-related theta power could not be linked to biased instrumental learning of Go/NoGo responses as reported in the main text.

**References**

1. Cohen MX, Wilmes K, van de Vijver I. Cortical electrophysiological network dynamics of feedback learning. Trends in Cognitive Sciences. 2011. pp. 558–566. doi:10.1016/j.tics.2011.10.004
