# Supplementary material for: Frontal network dynamics reflect neurocomputational mechanisms for reducing maladaptive biases in motivated action
Source: PLoS Biol. 2018 Oct 18;16(10):e2005979. doi: 10.1371/journal.pbio.2005979 (PMC6207318; doi:10.1371/journal.pbio.2005979)
Supplement: S6 Text — (DOCX) [file pbio.2005979.s006.docx]

**S6 Text. Midfrontal-motor phase synchrony.**

In the main text we reported our findings regarding midfrontal-motor phase synchrony. Specifically, we assessed whether midfrontal-motor ISPS was affected by motivational congruency, and whether this modulation depended on whether the motor site was associated with motor execution (contralateral Go) or no motor execution (ipsilateral Go or bilateral for NoGo). To elaborate, we reasoned that the motor cortex phase synchrony might behave differently for contra- and ipsilateral Go responses, as the contra- and ipsilateral motor cortex can be considered functionally different (i.e., the ‘executing’ vs. ‘non-executing’ motor site). Crucially, midfrontal functional connectivity has been proposed to be activity-dependent [1], such that target sites become more responsive to the midfrontal signals when they are more active. Thus, executing and non-executing motor sites might differ in midfrontal-motor phase synchrony due to differences in task activation. To be able to assess such lateralization, we adapted the experimental setup such that subjects now needed to respond with the left and right hand, whereas in our previous study subjects responded with the index and middle finger of one hand [2]. Finally, we reasoned that the motor cortex during NoGo responses would be most comparable to the ipsilateral motor cortex during Go responses, as these sites did not instantiate an overt Go response, and therefore grouped these together as the non-executing motor sites.

Following this line of reasoning, we started off with a non-directional ANOVA with the factors Valence x Required Response (Go_contra_ / Go_ipsi_ / NoGo_bilateral_), and after establishing a significant Valence x Required Response interaction (*F*_2,54_=4.0, *p*=.024), we continued with our planned contrast including the factors Congruence (congruent vs. incongruent) x Motor Execution (executing vs. non-executing) to assess whether the midfrontal-motor phase coherence showed a congruency effect for the executing and non-executing sites. Here we observed that ISPS with the non-executing motor increased with motivational incongruency (*F*_1,27_=5.3, *p*=.029), resembling the midfrontal-lateral prefrontal phase synchrony, whereas phase synchrony with the executing-motor cortex showed, if anything, a trend in the opposite direction (Congruency x Motor Execution: *F*_1,27_=10.2, *p*=.004); midfrontal-motor_contra_ ISPS was marginally higher for the Go-to-Win cues than the Go-to-Avoid cues (*t*_28_=1.7, *p*=.097). Thus, we observed that midfrontal-motor phase synchrony increased during motivational conflict for the non-executing motor sites, but, if anything, decreased for the executing (contralateral) motor sites.

The midfrontal-motor phase synchrony findings might be reconciled by considering activity-dependent functional connectivity. As mentioned above, it is thought that the midfrontal theta signals might be conveyed to all nodes in the network, whereas only active areas become susceptible to the midfrontal theta-band input [1], giving rise to activity-dependent functional connectivity. As such, we do not need to assume that the midfrontal cortex “knows” to which areas to send the information in order to implement control. Accordingly, control-related functional connectivity has been observed between the midfrontal cortex and several target sites, depending on the task at hand[3]. Consistent with these theories, we also observed a motivational conflict-related increase in phase synchrony between the midfrontal and lateral prefrontal sites, and between the midfrontal and motor sites that did not instantiate an overt motor response. Surprisingly, however, we observed the opposite (non-significant) pattern for the motor sites contralateral to active ‘Go’ responses. Phase synchrony between the midfrontal and contralateral motor sites was marginally stronger in reward than punishment contexts, even though active ‘Go’ responding can be considered motivationally congruent with reward contexts. Although we acknowledge that this effect for the executing motor sites was not significant (and we should therefore be cautious to interpret this effect), we would nevertheless like to provide the following potential explanation for this surprising observation; That is, these findings could be reconciled by considering that reward cues facilitate behavioral activation [4–6] through activation of the basal ganglia ‘Go’-pathways [7–9], increasing activation in the motor cortex [10]. The enhanced (reward-related) activation might have made the contralateral motor cortex more susceptible to the midfrontal theta-band signals, even though the midfrontal sites displayed weaker theta-band activation during the Go-to-Win trials. Of course such reward-related activation might also hold for the non-executing motor sites, but could nevertheless be most pronounced for the motor cortex that instantiates an overt motor response. To summarize, midfrontal-motor phase synchrony significantly increased with motivational conflict in the non-executing motor sites, but marginally decreased in the contralateral motor sites, potentially due to enhanced susceptibility for midfrontal connectivity resulting from reward-related activation of the contralateral motor cortex.

**References**

1. Cohen MX. A neural microcircuit for cognitive conflict detection and signaling. Trends in neurosciences. 2014. pp. 480–490. doi:10.1016/j.tins.2014.06.004

2. Swart JC, Froböse MI, Cook JL, Geurts DEM, Frank MJ, Cools R, et al. Catecholaminergic challenge uncovers distinct Pavlovian and instrumental mechanisms of motivated (in)action. Elife. 2017;6. doi:10.7554/eLife.22169

3. Cavanagh JF, Frank MJ. Frontal theta as a mechanism for cognitive control. Trends in Cognitive Sciences. 2014. pp. 414–421. doi:10.1016/j.tics.2014.04.012

4. Guitart-Masip M, Duzel E, Dolan R, Dayan P. Action versus valence in decision making. Trends in Cognitive Sciences. 2014. pp. 194–202. doi:10.1016/j.tics.2014.01.003

5. Dayan P, Niv Y, Seymour B, D. Daw N. The misbehavior of value and the discipline of the will. Neural Networks. 2006;19: 1153–1160. doi:10.1016/j.neunet.2006.03.002

6. Niv Y, Daw ND, Joel D, Dayan P. Tonic dopamine: Opportunity costs and the control of response vigor. Psychopharmacology (Berl). 2007;191: 507–520. doi:10.1007/s00213-006-0502-4

7. Collins AGE, Frank MJ. Opponent actor learning (OpAL): Modeling interactive effects of striatal dopamine on reinforcement learning and choice incentive. Psychol Rev. 2014;121: 337–66. doi:10.1037/a0037015

8. Hernandez-Lopez S, Bargas J, Surmeier DJ, Reyes A, Galarraga E. D1 receptor activation enhances evoked discharge in neostriatal medium spiny neurons by modulating an L-type Ca2+ conductance. J Neurosci. 1997;17: 3334–3342. Available: http://www.ncbi.nlm.nih.gov/pubmed/9096166

9. Hernandez-Lopez S, Tkatch T, Perez-Garci E, Galarraga E, Bargas J, Hamm H, et al. D2 dopamine receptors in striatal medium spiny neurons reduce L-type Ca2+ currents and excitability via a novel PLC[beta]1-IP3-calcineurin-signaling cascade. J Neurosci. 2000;20: 8987–8995. doi:20/24/8987 [pii]

10. Chiu Y-C, Cools R, Aron AR. Opposing Effects of Appetitive and Aversive Cues on Go/No-go Behavior and Motor Excitability. J Cogn Neurosci. 2014;26: 1851–1860. doi:10.1162/jocn_a_00585
