# Supplementary material for: Frontal network dynamics reflect neurocomputational mechanisms for reducing maladaptive biases in motivated action
Source: PLoS Biol. 2018 Oct 18;16(10):e2005979. doi: 10.1371/journal.pbio.2005979 (PMC6207318; doi:10.1371/journal.pbio.2005979)
Supplement: S7 Text — ISPS, intersite phase synchrony. (DOCX) [file pbio.2005979.s007.docx]

**S7 Text. Single trial power and intersite phase synchrony.**

In the main text, we showed that motivational conflict modulated trial-averaged midfrontal theta power and intersite phase synchrony with motor and prefrontal sites. Here we assessed whether the single-trial estimates could capture the main conflict modulations as observed at the trial-averaged level, before using the single-trial estimates in the computational models. Single-trial power and intersite phase synchrony (ISPS) estimates were analyzed using mixed-model regression analyses. These models included the within subject factors Valence (Win vs. Avoid) and Required Action (Go vs. NoGo) for the power and ISPS_midfrontal-prefrontal_ analysis. For the ISPS_midfrontal-motor_ analysis, we analyzed the conflict modulation again as a function of executing vs. non-executing motor side. Accordingly, the model included the within subject factors Congruency (congruent vs. incongruent) and Motor Execution (executing vs. non-executing), where the effect of Congruency translates to the Valence x Required Action interaction in the other models. All models included a full random effects structure; the power and ISPS values were mean-corrected and inverse transformed to improve normality.

Results of the single-trial analysis of midfrontal theta power were in line with the trial-averaged analyses. The single-trial power values increased under motivational conflict (X^2^(1)=8.7, *p*=.003), in the absence of main effects of Valence (X^2^(1)=2.7, *p*=.098) and Required Action (X^2^(1)=1.3, *p*=.971). The modulation by valence was significant for the NoGo cues (X^2^(1)=11.3, *p*<.001), and not for the Go cues (X^2^(1)=.9, *p*=.348).

In the main analyses we excluded incorrect trials to minimize contamination by error-related signals, since both conflict detection and error processing have been linked to increased midfrontal theta power[1,2]. The conflict detection signals commonly peak prior to the response, while the error processing signals peak after the response[2]. Although our time-window of interest (450-650ms) precedes the average response time (mean=753ms; range=594-1077ms), the power estimates might capture error processing signals nonetheless due to the temporal smoothing that is inherent in time-frequency analyses. We tested whether theta power increased on error trials, but observed the opposite pattern: the midfrontal theta power values increased for the correct trials relative to incorrect trials (X^2^(1)=17.1, *p*<.001), speaking against the contamination by error processing. Furthermore, motivational conflict did not significantly modulate the power estimates on incorrect trials (X^2^(1)=.2, *p*=.685), suggesting that midfrontal theta power particularly increases with motivational conflict when the conflict is correctly overcome. Note, however, that a mixed-level model with the full interaction (Valence x Required Action x Accuracy) did not converge. This is likely due to the low error rate particularly on congruent trials, leaving this interaction underpowered. To nonetheless assess this interaction, we circumvent this convergence issue by testing this interaction on the trial-averaged data using repeated measures ANOVA. This ANOVA indicated a significant three-way interaction (Valence x Required Action x Accuracy*: F*_1,26_=6.6, *p*=.016), driven by a significant interaction for the correct trials (Valence x Required Action: *F*_1,29_=9.6, *p*=.004) and a non-significant interaction for the incorrect trials (Valence x Required Action: *F*_1,26_=1.2, *p*=.286). Three subjects lacked EEG data for the incorrect NoGo-to-Avoid trials and were therefore not included in this analysis. Given the low error rate on the motivationally congruent trials for some subjects, these results should be interpreted with caution. Taken together, the single-trial power estimates showed the modulation by motivational conflict, particularly when the conflict was successfully overcome, and did not show an error-related increase. This pattern of results could suggest that subjects might not have detected the motivational conflict on the incorrect trials, leaving them to follow their prepotent, Pavlovian response tendencies. Therefore, we included the single-trial power estimates of both correct and incorrect trials in the computational modeling section.

Motivational conflict also modulated midfrontal-prefrontal phase synchrony at the single-trial level, such that the phase synchrony estimates increased with motivational conflict (X^2^(1)=5.4, *p*=.021). The phase synchrony modulation by motivational conflict showed only a significant simple effect for the NoGo cues (X^2^(1)=8.5, *p*=.003), and not for the Go cues (Χ^2^(1)=.3, *p*=.608). No other effects were significant (Valence: X^2^(1)=3.5, *p*=.062; Required Action: X^2^(1)<.1, *p*=.886).

The single-trial midfrontal-motor ISPS results again showed an effect of motivational conflict that depended on whether the motor site instantiated an overt Go response (Congruency x Motor Execution: X^2^(1)=4.1, *p*=.044). Thus, the midfrontal-motor ISPS increased with motivational conflict in the non-executing motor site (i.e. ipsilateral to Go responses and bilateral for NoGo responses), but decreased with motivational conflict in the executing motor site (i.e. contralateral to Go responses), although both simple effects were non-significant (non-executing: X^2^(1)=1.8, *p*=.176; executing: X^2^(1)=2.0, *p*=.158). Furthermore, single-trial midfrontal-motor phase synchrony increased for Win relative to Avoid cues across responses (X^2^(1)=4.1, *p*=.042). Single-trial midfrontal-motor phase synchrony did not significantly differ between the motor sites during NoGo responses and contralateral Go responses (X^2^(1)=1.0, *p*=.329), or ipsilateral Go responses (X^2^(1)=.2, *p*=.679). Altogether, the single-trial estimates for both power and intersite phase synchrony replicate the modulations by motivational conflict as observed at the trial-averaged level.

As we include the power and phase synchrony measures in competing computational models (family M4 and M5 respectively), we assessed how correlated the trial-by-trial estimates are, in order to assess whether there is sufficient unexplained variance between these measures. To this end, we computed the within-subject correlations between trial-by-trial midfrontal theta power and i) midfrontal-lateral prefrontal phase synchrony, and ii) midfrontal-motor phase synchrony per condition (see Fig S1). The average correlation between midfrontal theta power and midfrontal-lateral prefrontal phase synchrony was .38 (range: .22 to .62), and the average correlation for midfrontal theta power and midfrontal-motor phase synchrony was .32 (range: .12 to .53). Thus, even for the highest observed correlation (R=+.62; R^2^=.38), there was still 62% unexplained variance between the power and phase synchrony measures. In other words, despite clear covariation between the power and phase synchrony measures, there was extensive unique variance.


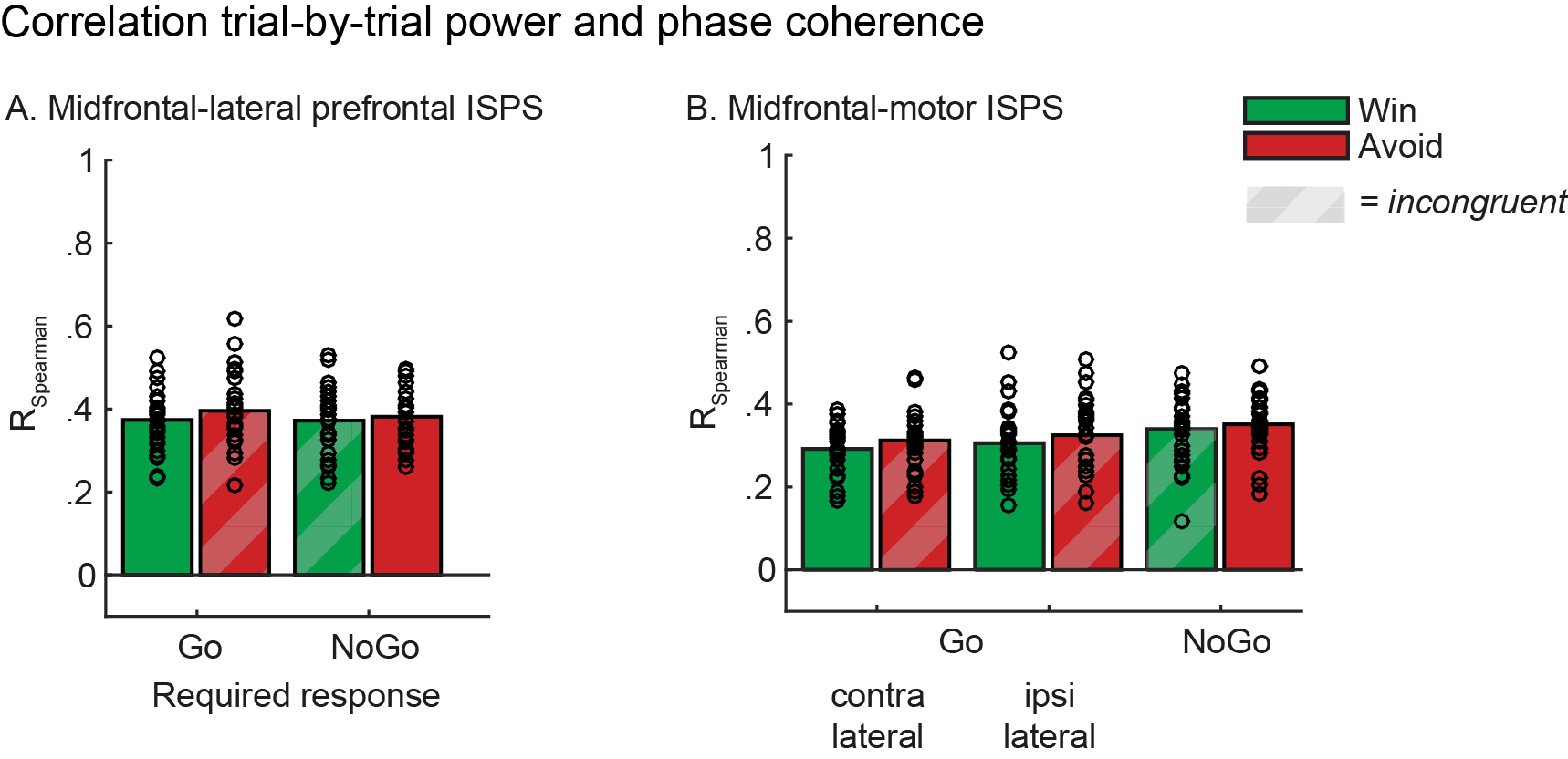


Fig S1. Correlations between single-trial midfrontal theta power and intersite phase synchrony.

(a) Correlations for trial-by-trial midfrontal theta power and midfrontal-lateral prefrontal phase synchrony per subject (black circles), ranging from +.22 to +.62, where the lowest observation of unexplained variance still reaches 62%. (b) Correlations for trial-by-trial midfrontal theta power and midfrontal-motor phase synchrony per subject, ranging from +.12 to +.53. Underlying data can be found at http://hdl.handle.net/11633/di.dccn.DSC_3017033.03_624.

**References**

1. Cavanagh JF, Frank MJ. Frontal theta as a mechanism for cognitive control. Trends in Cognitive Sciences. 2014. pp. 414–421. doi:10.1016/j.tics.2014.04.012

2. Cohen MX. A neural microcircuit for cognitive conflict detection and signaling. Trends in neurosciences. 2014. pp. 480–490. doi:10.1016/j.tins.2014.06.004
