# Supplementary material for: Frontal network dynamics reflect neurocomputational mechanisms for reducing maladaptive biases in motivated action
Source: PLoS Biol. 2018 Oct 18;16(10):e2005979. doi: 10.1371/journal.pbio.2005979 (PMC6207318; doi:10.1371/journal.pbio.2005979)
Supplement: S8 Text — ISPS, intersite phase synchrony. (DOCX) [file pbio.2005979.s008.docx]

**S8 Text. Computational modelling: potential alternative mechanisms related to intersite phase synchrony.**

In the analyses described in the main text, we assumed that the phase synchronization of the task-relevant clusters to the midfrontal cluster would impact the same system as local midfrontal theta power (M5). Alternatively, the phase synchronization might have target-dependent impact, where the lateral prefrontal synchrony might relate to the modulation of the goal representations (in this task, the instrumental action values), whereas midfrontal-motor synchrony might relate to modulation of the motor excitability. We tested these alternative mechanisms in a new set of models, M6. We used the same intersite synchrony measures, but now allowed the midfrontal-prefrontal synchrony to scale the impact of the instrumental controller (M6a; cf. Eq. 7) and midfrontal-motor synchrony to scale the contralateral action weight (M6b):

$w\left( {Go'}_{t},s_{t} \right)= \left\{ \begin{matrix} Q\left( {Go'}_{t},s_{t} \right)+\pi V\left( s \right)+ b+ \beta*I{SPS}_{motor,contra} if conflict \\ Q\left( {Go'}_{t},s_{t} \right)+ \pi V\left( s \right)+ b else \end{matrix} \right.$

Model evidence reduced relative to the M5 models for these alternative synchrony models, where midfrontal-lateral prefrontal synchrony modulated the instrumental contribution (WAIC_M6a_=28615), and midfrontal-motor synchrony modulated motor excitability (WAIC_M6b_=28557). Altogether, model comparison favored synchrony models where midfrontal-lateral prefrontal (WAIC_M5a_=28477) and midfrontal-motor phase synchrony (WAIC_M5b_=28425) modulated the Pavlovian bias. These findings are in line with the proposal that the midfrontal cortex signals the need to adjust the decision-threshold to the task-related network, in order to prevent impulsive, Pavlovian responses.
